# Association between single-nucleotide polymorphisms in endochondral development-related genes and 3D phenotypic variation of the cranial base

**DOI:** 10.1186/s13005-026-00644-8

**Published:** 2026-07-21

**Authors:** Guido Artemio Marañón-Vásquez, Mônica Tirre de Souza Araújo, Antônio Carlos de Oliveira Ruellas, Mírian Aiko Nakane Matsumoto, Alejandro David Avalos Chávez, Marcio Figueiredo, Thaís de Oliveira Fernandes, Lívia Azeredo Alves Antunes, Manuel Lagravère Vich, Rafaela Scariot, Carlos Flores-Mir, Christian Kirschneck, Leonardo dos Santos Antunes, Erika Calvano Küchler

**Affiliations:** 1 Department of Pediatric Dentistry, School of Dentistry of Ribeirão Preto, University of São Paulo, Avenida do Café, s/n, São Paulo 14040-904 Ribeirão Preto, Brazil; 2 Department of Pediatric Dentistry and Orthodontics, School of Dentistry, Federal University of Rio de Janeiro, Rua. Prof. Rodolpho Paulo Rocco, 325 – Cidade Universitária da Universidade Federal do Rio de Janeiro, Rio de Janeiro, RJ 21941-617 Brazil; 3 Department of Specific Formation, School of Dentistry, Fluminense Federal University, Rua. Dr. Silvio Henrique Braune, 22 - Centro, Nova Friburgo, Rio de Janeiro 28625-650 Brazil; 4 School of Dentistry, Faculty of Medicine and Dentistry, University of Alberta, 5-524, 11405 87 Ave NW, Edmonton, AB T6G 1C9 Canada; 5 Department of Stomatology, School of Dentistry, Federal University of Paraná, Av. Prefeito Lothário Meissner, 632 - Jardim Botânico, Curitiba, PR 80210-170 Brazil; 6 Department of Orthodontics, University Hospital of Bonn, Welschnonnenstr. 17, Bonn, 53111 Germany

**Keywords:** Skull Base, Polymorphism, Single Nucleotide, BMP2 protein, human, BMP4 protein, human, RUNX2 protein, human, Core Binding Factor Alpha 1 Subunit, Smad6 Protein

## Abstract

**Background:**

This study aimed to evaluate the association between single nucleotide polymorphisms (SNPs) in endochondral development-related genes and cranial base 3D phenotypes.

**Methods:**

CBCT scans and the genomic DNA of 118 individuals were evaluated (age range: 15–66 years; 82 females). Data from eleven 3D landmarks identified at the cranial base were subjected to geometric morphometric analysis, including Procrustes fit, principal component (PC) analyses, and estimation of centroid sizes and fluctuating asymmetry scores. Seven SNPs within *BMP2*, *BMP4*, *RUNX2*, and *SMAD6* were genotyped by real-time PCR. General linear models (GLM) were fitted to assess the effect of SNPs on cranial base shape, size, and symmetry quantitative traits.

**Results:**

Seven PCs were identified for each shape variation aspect (i.e., symmetric and asymmetric components). These explained 81.9% and 84.6% of the total variation, respectively. GLM did not evidence strong associations between cranial base shape aspects and the studied SNPs. GLM including age, sex and *BMP2* rs1005464 as predictor variables, showed a strong explanatory power of the variation in the size of the cranial base (adjusted R^2^ = 0.50). Individuals carrying at least one A allele for rs1005464 had larger cranial bases than common GG homozygotes (β = 0.015; 95% CI: 0.003, 0.027; *P* = 0.016). Fluctuating asymmetry scores were not associated with any of the evaluated SNPs.

**Conclusions:**

The results suggest that *BMP2* rs1005464 could be associated with variations in the cranial base size.

**Supplementary Information:**

The online version contains supplementary material available at 10.1186/s13005-026-00644-8.

## Background

The cranial base plays a critical role in the integration of overall craniofacial development [1]. Endochondral ossification grows this structure with a well-recognized influence of intrinsic genetic factors [2–5]. Signaling pathways that regulate the formation and growth of long bones might be involved in developing the cranial base [3, 6, 7].

Bone morphogenetic proteins (BMPs), a relevant group of phylogenetically conserved growth factors, are expressed at different stages of cranial base pre- and postnatal growth [8]. The BMP signaling, including BMP2 and BMP4, would play regulatory roles in early development, chondrocyte proliferation and differentiation, and ossification of this structure [8, 9]. Mothers Against Decapentaplegic Homolog 6 (SMAD6) inhibits BMP signaling by interacting with transcription repressors [10]. This protein modulates several stages of chondrogenesis by BMP-mediated regulation of Ihh expression and activity [11]. Runt-related Transcription Factor 2 (RUNX2) is another molecule with an important role during chondrogenesis that is also expressed in cranial base synchondroses and adjacent regions [12]. It has been demonstrated that up-regulation of RUNX2, secondary to *Tbx1* or *Hdac4* deficiency, generates early ossification of these structures [13, 14].

Some single nucleotide polymorphisms (SNPs) in *BMP2*, *BMP4*, *RUNX2* and *SMAD6* were previously associated with malocclusion-related traits in humans [15–17]. Considering that these genes encode molecules with a relevant role in cranial base endochondral development and that configuration and size of this structure, in turn, would influence the presence of malocclusions [18, 19], it is reasonable to hypothesize that SNPs in the mentioned genes could either have (a) a primary effect on cranial base or (b) that a pleiotropic effect on different craniofacial structures occurs. From this perspective, variants in *BMP2*,* BMP4*,* RUNX2* and *SMAD6* would constitute potential candidates to investigate the involvement of these genes in the phenotypic variability of the cranial base.

Based on the above, the present study aimed to evaluate phenotype-genotype relationships between cranial base traits (i.e., 3D aspects of shape, size, and symmetry) and SNPs in *BMP2*, *BMP4*, *RUNX2*, and *SMAD6*.

## Methods

The Research Ethics Committee of the School of Dentistry of Ribeirão Preto, University of São Paulo, approved the protocol of this study (n. 3.150.551). Unrelated healthy individuals from private and graduate dental clinics in São Paulo, Brazil, were assessed and selected as part of convenience sampling. The main eligibility criterion was to have a pre-treatment cone beam computed tomography (CBCT) scan covering the cranial base’s full extent. CBCT of all the studied subjects were taken for clinical purposes. Individuals who have not completed the pubertal growth spurt of the jaws (< 15 years old) [20], with known syndromes or dentofacial anomalies, a history of facial trauma, orthopaedic and / or ortho-surgical treatment, or poor-quality CBCT images were not included. The sample consisted of 118 individuals (age range: 15–66 years; mean age = 32.1 ± 14.4 years; 36 males, 82 females; skeletal Class I [0° < ANB < 4°]: *n* = 39, Class II [ANB ≥ 4°]: *n* = 45, Class III [ANB ≤ 0°]: *n* = 34). Signed written informed consent was obtained from all participants and / or legal guardians at the beginning of the study.

### Phenotyping

**Fig. 1:**
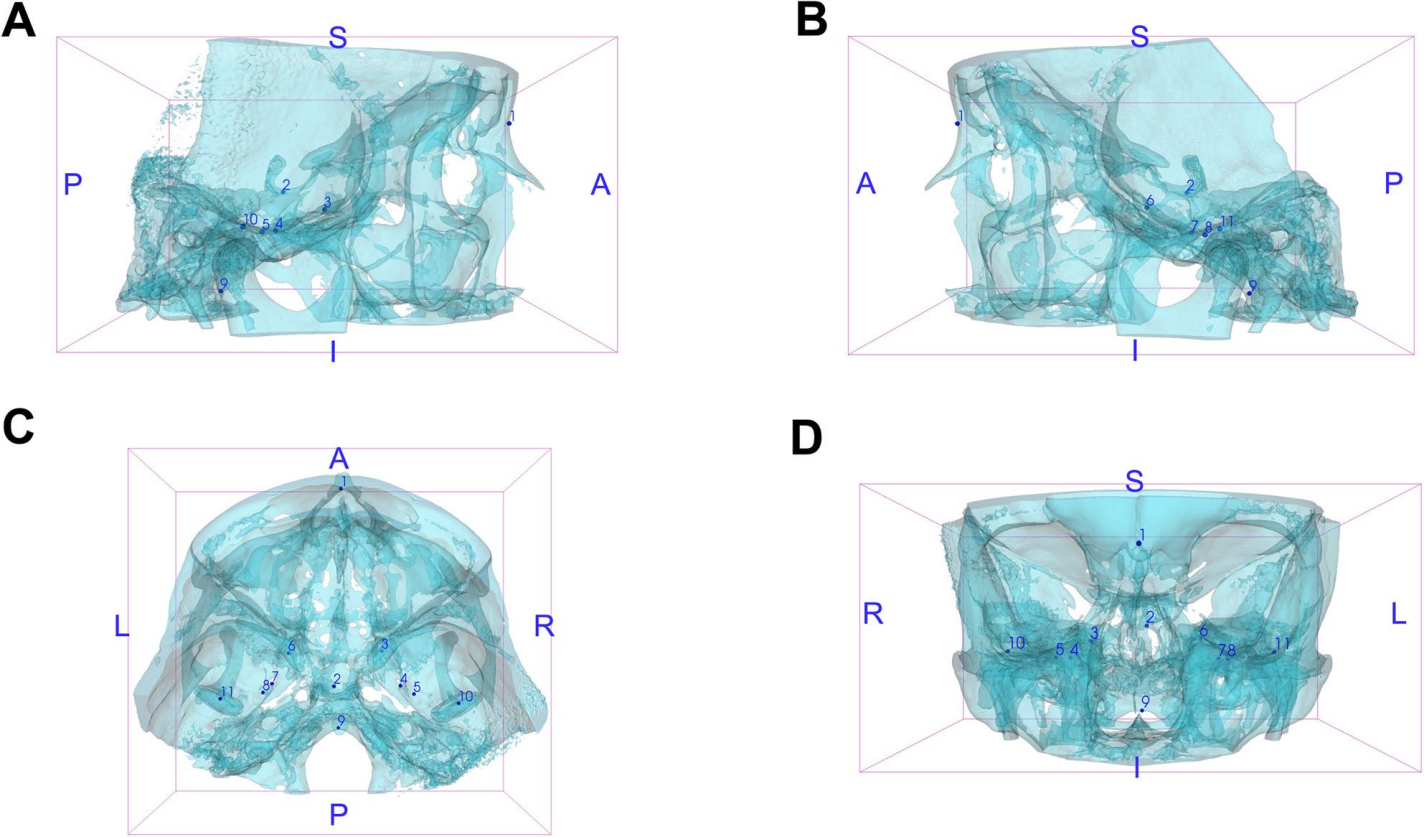
Cranial base 3D landmarks. **A** (black letter) – right side view; **B** – left side view; **C** – top view; **D** – anterior view; S – superior; I - inferior; A (blue letter) – anterior; P – posterior; R – right; L – left; 1 – Nasion; 2 – inferior Sella; 3 and 6 – right and left foramen Rotundum, respectively; 4 and 7 – right and left foramen Ovale, respectively; 5 and 8 – right and left foramen Spinosum, respectively; 9 – Basion; 10 and 11 – right and left Glenoid fossa, respectively

Twenty-five 3D landmarks on the cranial base (Table 1) were initially assessed on CBCTs using open-source software ITK-SNAP 3.6.0 (http://www.itksnap.org) and 3D Slicer 5.0.3 (http://www.slicer.org). Preliminarily, scans were resampled to an isotropic voxel size of 0.25 mm and were oriented to match the sagittal plane with N, CG and Ba and the axial plane with the Frankfurt horizontal plane. As previously described [21], using ITK-SNAP, landmarks were pre-labelled on 3D reconstructions based on semi-automatic segmentations of the cranium and using the three cross-sectional views as a reference to improve the reliability of landmarks placement. Then, in 3D Slicer, 3D surface models were generated from pre-labelled segmentations, where definitive landmarks were located over the landmarks previously placed in ITK-SNAP. After evaluating the entire sample, 14 landmarks were excluded either because they were outside the field of view of the images (CG; PFM; and, right and left HC, LFM and EAM) or significant anatomical variability, making it impossible to consistently identify them (right and left ACP, PCP and JF) in more than 10% of the sample. Finally, 11 3D landmarks were included in the subsequent analyses (Fig. 1).

**Table 1:** Definition of 3D landmarks

Landmark	Location
Nasion (N)	The most anterior point of the Fronto-nasal suture at the midsagittal plane.
Crista Galli (CG)	The most superior point of the apophysis Crista Galli.
Inferior Sella (IS)	The lowest point on the Pituitary fossa at the midsagittal plane.
Anterior Clinoid process (ACP)*	The most posterior point of the anterior Clinoid process.
Posterior Clinoid process (PCP)*	The most anterior point of the posterior Clinoid process.
Foramen Rotundum (FR)*	Central point of the foramen Rotundum on the side of the cranial vault, when its circumference is completed in the coronal view.
Foramen Ovale (FO)*	Central point of the foramen Ovale when its circumference is completed in the axial view.
Foramen Spinosum (FS)*	Central point of the foramen Spinosum when its circumference is completed in the axial view.
Jugular foramen (JF)*	The most anterior point (most constricted region) of the Jugular foramen on the side of the cranial vault.
Hypoglossal canal (HC)*	Central point of the medial meatus of the Hypoglossal canal when its circumference is completed in the coronal view.
Lateral border of the foramen Magnum (LFM)*	The most lateral point of the curvature of the side of the foramen Magnum at the most convex point of the margin of the bone.
Basion (Ba)	The most inferior point of the anterior margin of the foramen Magnum at the midsagittal plane.
Posterior contour of the foramen Magnum (PFM)	The most inferior point of the posterior margin of the foramen Magnum at the midsagittal plane.
External auditory meatus (EAM)*	The most superior point of the external auditory meatus.
Glenoid fossa (GF)*	The most superior point of the Glenoid fossa.

*Paired landmarks located on the right and left sides

Twenty percent of the sample was randomly selected and evaluated a second time by the same researcher after at least two weeks to assess the method error. Repeated 3D coordinate data of the selected landmarks were assessed for intra-observer repeatability, and random and systematic errors utilizing the intraclass correlation coefficient (ICC), Dahlberg’s formula and the Bland-Altman method (proportion bias), respectively. The evaluations evidenced high repeatability (ICC ranging from 0.79 to 0.99), reasonable measurement error (X-axis: 0.10–0.58 mm, Y-axis: 0.20–0.70 mm, Z-axis: 0.11–0.44 mm), and absence of proportion bias (Additional file: Table S1).

**Fig. 2:**
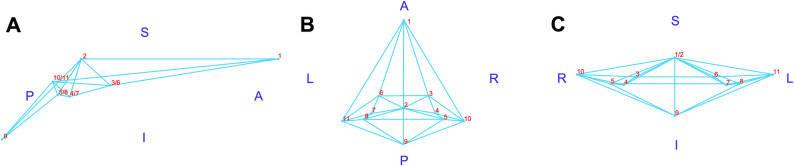
Wireframes for the average configuration of cranial base 3D landmarks. **A** (black letter) – X vs. Y axis (right side view); **B** – X vs. Z axis (top view); **C** – Y vs. Z axis (anterior view); S – superior; I - inferior; A (blue letter) – anterior; P – posterior; R – right; L – left; 1 – Nasion; 2 – inferior Sella; 3 and 6 – right and left foramen Rotundum, respectively; 4 and 7 – right and left foramen Ovale, respectively; 5 and 8 – right and left foramen Spinosum, respectively; 9 – Basion; 10 and 11 – right and left Glenoid fossa, respectively

Coordinate data of cranial base 3D landmarks were imported into the software MorphoJ 1.07a (https://morphometrics.uk/MorphoJ_page.html) to perform geometric morphometric analyses under object symmetry perspective [22, 23]. Data were submitted to full Procrustes fit, generating data matrices for symmetric and asymmetric components of variation. Covariance matrices were then created based on the new output data. Principal component (PC) analyses were run to identify the main aspects of shape variation (i.e., PCs explaining at least 5% of variation). Wireframes were created for the graphical representation of the PCs (Fig. 2). The size of the cranial base was evaluated via the estimation of the centroid size. Individual fluctuating asymmetry scores in units of Mahalanobis distances measured shape asymmetry. Data were subjected to Procrustes ANOVA to assess the individual and side effects on cranial base shape variation.

### Genotyping

As previously described, participants’ genomic DNA was extracted from buccal cells [24]. The studied SNPs were selected based on previous evidence suggesting an association with craniofacial phenotypes and their location on genes with biological activity in cranial base development. Seven SNPs across four genes (Table 2) were blindly genotyped by PCR using Endpoint analyses [25] and TaqMan chemistry in a real-time PCR system (Step One Plus Real-Time PCR System, Applied Biosystems^®^, Foster City, CA, USA). 10% of the sample was genotyped in duplicate, showing a 100% concordance rate. All SNPs had 100% genotyping rates, showed allele frequencies in Hardy-Weinberg equilibrium, and frequency of the minor allele > 15%.

**Table 2:** Single nucleotide polymorphisms studied

Gene	Locus	Reference sequence	Type of alteration	Base change (Context sequence)	MAF (ALFA)
*BMP2*	20p12.3	rs1005464	Intron variant	ATC[A/G]CCT	A = 0.2194
*BMP2*	20p12.3	rs235768	Missense variant*	CAG[A/T]CTT	A = 0.3678
*BMP4*	14q22.2	rs17563	Missense variant**	ATC[A/G]CCT	A = 0.4803
*RUNX2*	6p21.1	rs59983488	Intron variant	GGG[G/T]AGT	T = 0.0710
*RUNX2*	6p21.1	rs1200425	Intron variant	TTT[A/G]GAA	A = 0.3891
*SMAD6*	15q22.31	rs2119261	Intron variant	CTC[C/T]ATG	T = 0.3700
*SMAD6*	15q22.31	rs3934908	Intron variant	AAG[C/T]CCT	T = 0.4498

Sources of information: dbSNP from: http://www.ncbi.nlh.nih.gov/snp/; http://genome.uscs.edu/; and, https://www.thermofisher.com

*MAF *Minor allele frequency, *ALFA *Allele Frequency Aggregator (NCBI database of Genotypes and Phenotypes [dbGaP])

* Arg → Ser

** Val → Ala

### Statistical analysis

General linear models were fit by the ordinary least squares method to assess the effect of SNPs on the following cranial base quantitative traits: PC scores for symmetric and asymmetric components of shape variation, centroid size, and shape fluctuating asymmetry scores. The sex and age of participants were considered as covariates in the models. Analyses in the dominant and recessive models were also performed. A *P* value less than or equal to 0.05 was set as significant at the nominal level. The Benjamini-Hochberg procedure further adjusted the *P* values to decrease the false discovery rate due to multiple testing. All analyses were performed in Jamovi 2.3 software (https://www.jamovi.org/). Post-hoc estimates of the power achieved by the analyses were performed in G*Power 3.1.9.6.

## Results

**Fig. 3:**
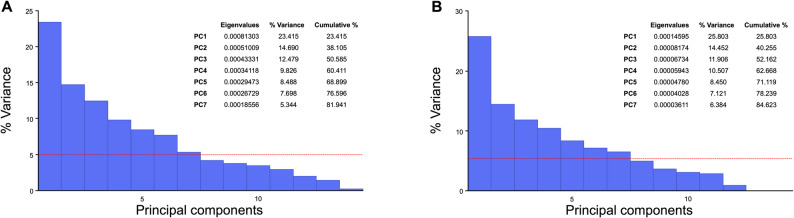
Scree plots showing the variance explained by symmetric (**A**) and asymmetric (**B**) PCs

**Fig. 4:**
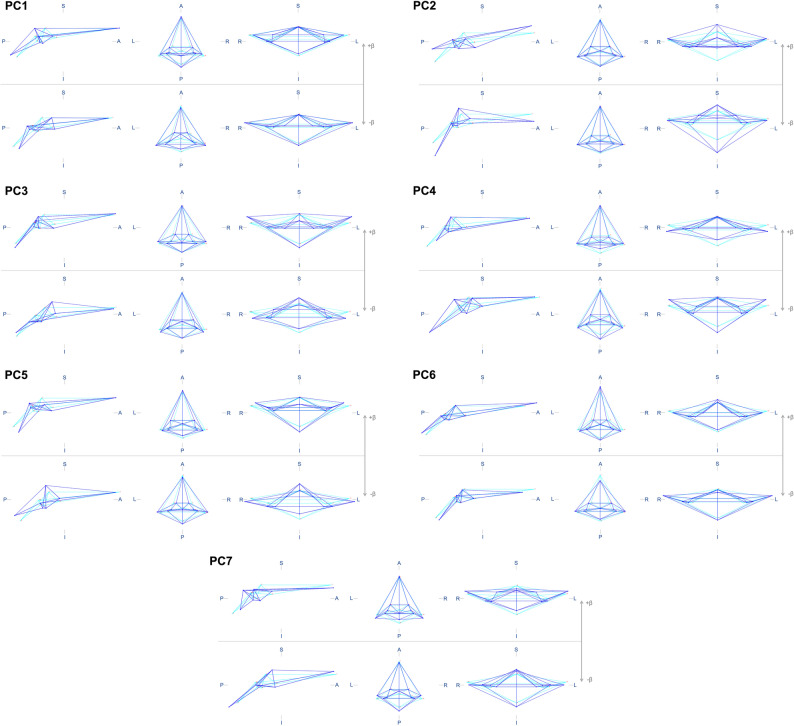
Cranial base shape configurations of subjects with the most negative (-β) or positive (+β) individual scores for symmetric PCs. S – superior, I - inferior, A – anterior, P – posterior, R – right, L – left. Green lines represent the average configuration, while blue lines represent the variation of interest

**Fig. 5:**
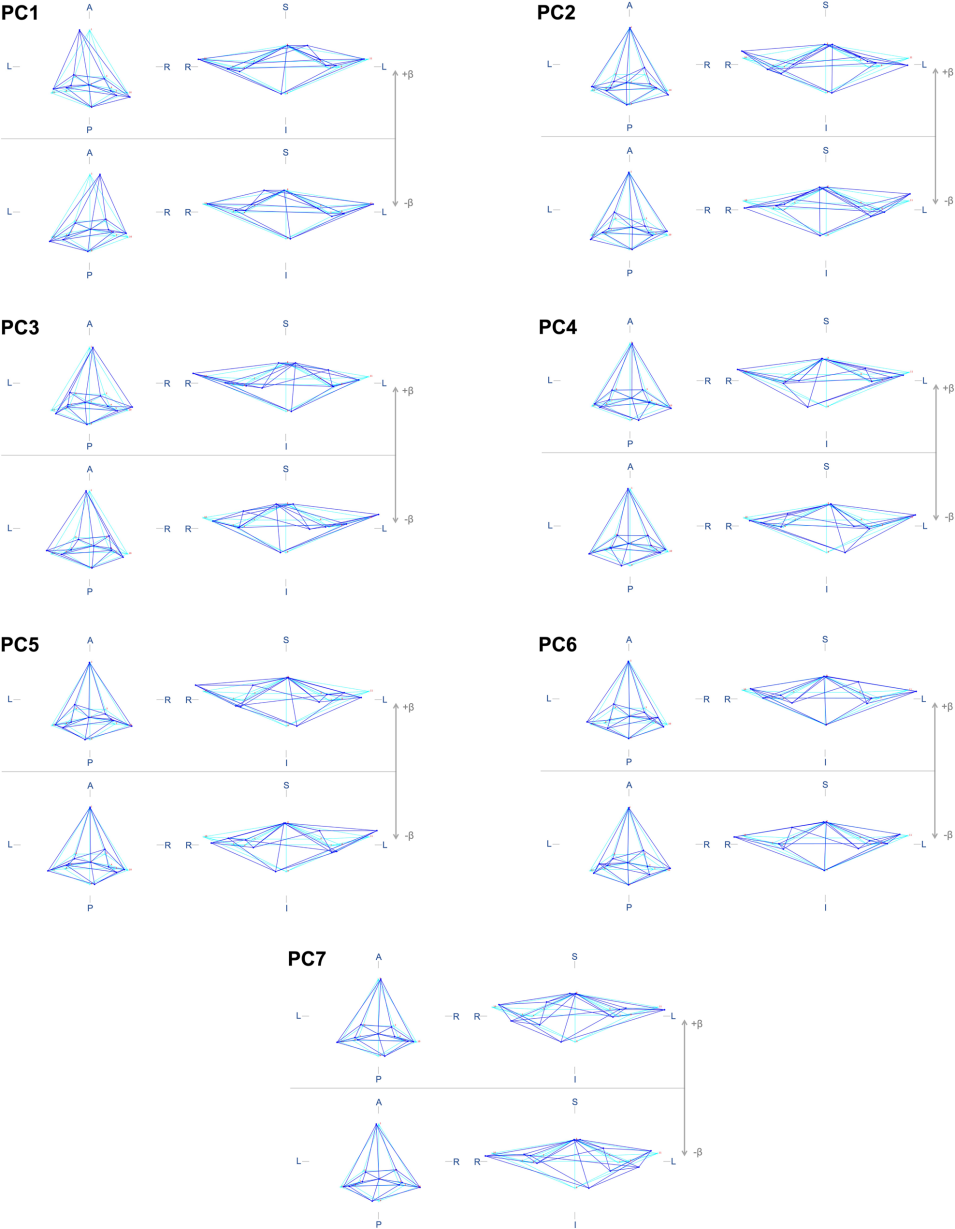
Cranial base shape configurations of subjects with the most negative (-β) or positive (+β) individual scores for asymmetric PCs. S – superior, I - inferior, A – anterior, P – posterior, R – right, L – left. Green lines represent the average configuration, while blue lines represent the variation of interest

PC analyses revealed seven components of symmetric and seven of asymmetric cranial base shape variation, which explained 81.9% and 84.6% of the total variation, respectively (Fig. 3). Symmetric components mainly represented relative shape variation in the following characteristics: PC1 - relative anteroposterior elongation versus transverse width of the cranial base, together with cranial base flexion; PC2 - cranial base flexion; PC3, PC4 and PC7 - relative anteroposterior configuration of the anterior and posterior cranial bases and the 3D configuration of the temporal bones; PC5 and PC6 – relative transverse relationships between the sphenoid and temporal bones. Asymmetric components mainly depicted variations in the following features: PC1, PC4, PC5 and PC7 - side orientation of the anterior or posterior cranial base, with or without the involvement of 3D configuration of the sphenoid and temporal bones; PC2 and PC6 - right-left difference in the sagittal and vertical components of the sphenoid and / or temporal bones; PC3 – the right-left difference in the transverse and vertical component of the sphenoid and temporal bones. Wireframes of the PCs are graphically shown in Figs. 4 and 5. Additional file: Table S2 describes detailed description of PC’s cranial base shape configurations, varying from those representing more negative to more positive individual scores. Procrustes ANOVA evidenced significant individual and side effects on cranial base 3D shape (*P* < 0.0001).

The complete results of the implemented statistical models for genetic evaluations are presented in Additional file: Table S3. The following phenotype-genotype relationships were significant at the nominal level (*P* < 0.05; F test *P* value < 0.05; Table 3): *BMP2* rs1005464 - centroid size, *BMP2* rs235768 - symmetric PC4 and PC6, and *BMP4* rs17563 - symmetric PC3. None of these associations remained significant after the Benjamini-Hochberg adjustment. Symmetric PC1, PC2, PC5 and PC7; asymmetric PC1 - PC7; and shape fluctuating asymmetry scores were not significantly influenced by the studied SNPs. Linear models on the effect of *BMP2* rs1005464 on centroid size evidenced the most substantial explanatory power (adjusted R^2^ = ~ 50%). Individuals carrying at least one A allele for *BMP2* rs1005464 had larger cranial bases than common GG homozygotes (β = 0.015; 95% CI: 0.003, 0.027; *P* = 0.016; adjusted R^2^ = 0.50; power = 0.99, Table 3).

**Table 3:** Single nucleotide polymorphisms with significant effects on cranial base phenotypes at the nominal level (*P* < 0.05)

Cranial base phenotypes	Gene SNP (1/2)*	Genotypes	Model coefficients				B-H *P* value	Model fit measures		Power estimate
			β	95% CI		*P* value		F test *P* value	Adjusted *R*^2^	
				lower	upper					
Symmetric PC3	*BMP4* rs17563 (G/A)	AG vs. AA	0.012	0.003	0.021	0.012	0.084	0.017	0.077	0.720
		AG + GG vs. AA	0.011	0.002	0.019	0.017	0.119	0.010	0.078	0.881
Symmetric PC4	*BMP2* rs235768 (A/T)	AT vs. TT	0.008	0.000	0.015	0.029	0.203	< 0.001	0.135	0.982
		AT + AA vs. TT	0.008	0.000	0.014	0.027	0.189	< 0.001	0.143	0.988
Symmetric PC6	*BMP2* rs235768 (A/T)	AA vs. AT + TT	-0.011	-0.022	0.000	0.048	0.336	< 0.001	0.147	0.990
Log centroid size	*BMP2* rs1005464 (A/G)	AG vs. GG	0.014	0.000	0.026	0.036	0.252	< 0.001	0.498	0.999
		AG + AA vs. GG	0.015	0.003	0.027	0.016	0.112	< 0.001	0.501	0.999

Common homozygous genotypes were established as the reference category in the models

*SNP *single nucleotide polymorphism, *CI *confidence interval, *B-H *Benjamini-Hochberg procedure, *PC *principal component, *Log *Natural logarithm

*(1 = minor allele / 2 = major allele)

The age of the participants significantly influenced symmetric (+β) and asymmetric (-β) PC4 scores, while sex had a significant effect on symmetric PC6 scores (+β) and centroid sizes (-β). Women had significantly smaller cranial bases than men. Skeletal malocclusion did not show an influence on the evaluated traits.

## Discussion

Information on specific SNPs influencing cranial base traits is limited. To our knowledge, this is the first genetic association study that broadly explored the phenotypic variability of the cranial base and evaluated the association with SNPs in endochondral development-related genes. Our results suggest a possible association between rs1005464 in *BMP2* and the size of the cranial base size.

Although the dynamic biomechanical mechanisms that occur during cranial base growth are well known [26], shape configurations that bones can present by the interplay of these mechanisms are still unclear. The present morphometric analyses revealed complex 3D configurations of this structure. In addition to the already reported variations in the cranial base angle and sagittal length, it was observed, as one of the main findings, that areas of the sphenoid bone that articulate with the middle third of the face, and the position of the glenoid fossae can assume distinct configurations regardless of the cranial base flexion/extension. This partially explains controversies on the relationship between bidimensional sagittal cranial base measures and the presence of malocclusions [18, 19].

Additionally, the results highlight two other interesting aspects: the variation in the relationship between the temporal and sphenoid bone’s transverse widths, and different right-left shape asymmetry configurations. Although the information on this subject is minimal, these findings could trigger studies about the cranial base as the structural foundation or contributing factor for developing different dentofacial alterations (e.g., transverse malocclusions, facial asymmetries). Nevertheless, extensive research is still needed to elucidate the real influence of cranial base 3D configuration on the facial skeleton, and possible compensatory morphological mechanisms between the different craniofacial structures.

Regarding the genetic analyses, rs1005464 in *BMP2* was significantly associated with the cranial base size. In general, it has been previously suggested that BMPs are molecules with a relevant role in regulating cartilage and bone formation [27]. The expression of the Bmp receptors in the cranial base anlagen suggests that BMP signaling also acts on this structure [28]. Specifically, *Bmp2* expression has been identified in early mesenchymal condensations during cranial base development [8]. Bmp signaling, including Bmp2, regulates *Sox9*, a crucial molecule for forming these condensations and subsequent chondrogenesis [29]. Interestingly, Bmp2 signaling seems to regulate the size and shape of limb mesenchymal condensations [30–32]. Similarly to long bones [31, 33], *Bmp2* is also expressed in the perichondrium of sites or nearby sites that contain osteoblastic cells, suggesting an additional role in the ossification process of this structure [8].

The association between *BMP2* rs1005464 and other traits (i.e., mandibular retrognathism, tooth size, dentoalveolar size discrepancies) has already been reported [15, 34–36]. Our findings complement the suggestion of a pleiotropic effect of this SNP on size-related craniofacial phenotypes. Additionally, a possible effect of *BMP2* rs235768 on shape patterning of the cranial base was observed. Despite the low explanatory significance of the implemented linear models, this variant was associated with cranial base shape configurations identified for the present sample (i.e., PC4 and PC6). Previous studies have also shown a relationship of rs1005464 or rs235768 in *BMP2* with shape variations of other craniofacial structures (i.e., root curvature of incisors, root canal shape, palatal rugae morphology) [37–39]. All the aforesaid evidence, together with the molecular information mentioned above, support the role that *BMP2* would have in the patterning of size and shape of craniofacial structures, including the cranial base.

As a complex trait, the cranial base configuration, is probably influenced by a plethora of environment and gene additive effects and / or epistatic interactions; therefore, several other SNPs could influence the assessed phenotypes. It should be emphasized that the absence of association of the other SNPs evaluated does not rule out the involvement of *BMP4*, *RUNX2* and *SMAD6* on the cranial base configuration. It is likely that different variants in these genes, perhaps not yet identified, may have a relevant effect. Some additional genes with a critical role in the formation, growth and development of the cranial base (e.g., genes involved in signaling pathways of the *BMP*, *FGF/FGFR*, *SHH*, *SOX9*, *WNT* families; *PTHrP*; *MSX2*; *FOXC1*; *SIX2*; *TBX1*, *IHH*, *EVC/EVC2*, *DDRs*) [3, 6, 7], as well as genes related to the asymmetry of dentofacial traits (e.g., *BMP3*, *ENPP1*, *ESR1*, *LATS1*, *SATB2*) [40, 41], would be interesting additional candidates for new related genetic association studies. Genome-wide association studies or candidate genes approach investigations are suggested to search for clinically relevant variants in other populations.

The sex and age of the participants had significant contributions to some of the statistical models implemented in this study. Sex was associated with variations in the shape and size of the cranial base. Previous evidence corroborates this finding and reports sexual dimorphism in the shape and size of the brain and surrounding structures [42]. The age of the present study participants showed an influence on cranial base shape variations. The orthodontic literature has shown that some areas, including the anterior and posterior cranial bases, suffer dimensional alteration even into late adulthood [43, 44]. Although our sample included individuals without significant growth (i.e., over 15 years), symmetrical PC4, related to discrepancies between the relative dimensions of the anterior and posterior cranial base and variations in the vertical location of the glenoid fossae, was influenced by age, confirming that dimensional changes could occur throughout life concerning these aspects.

It is important to mention that this research has some limitations. The sample mainly included Class II patients, females, with a wide variation in age. Despite having included age and sex as covariates in the analyses, and although malocclusions did not influence the evaluated traits, there may be confounding due to unknown factors not controlled by the convenience sampling. Therefore, selection bias cannot be ruled out. Another limitation is the possibility of population stratification. Genetic association studies may be affected by differences in ancestry among participants, which can generate spurious phenotype-genotype associations when phenotypic traits and allele frequencies vary across subpopulations. Although the present sample was recruited from a relatively homogeneous geographic region, genetic ancestry was not assessed, and ancestry-informative markers were not included in the analyses. Therefore, residual confounding due to population structure cannot be ruled out. In another aspect, the study evaluated a limited number of SNPs. Moreover, all the reported associations did not remain significant after applying the Benjamini-Hochberg adjustment; consequently, the results should be cautiously assessed due to the possibility of type 1 error.

On the other hand, one of the study’s strengths is that, despite not having performed an a priori sample size calculation, the implemented models reached a power above 80% in most analyses. Another strength is the implemented method for the evaluation of the cranial base. Morphological assessment of this structure has commonly been restricted to bidimensional linear or angular measurements with limited information regarding its 3D configuration [18, 19]. The success of genetic studies assessing complex traits depends on accurately characterizing the phenotypes [45], so methods are needed to evaluate the cranial base comprehensively. In that sense, it has been suggested that landmark-based geometric morphometric analyses are more informative for detecting shape variation of complex structures than methods evaluating separate cephalometric measurements [46]. A suggestion for further research is that even more detailed analyses could be implemented incorporating additional regions of the cranial base. A larger sample size would be necessary for consistent PC analyses, including more landmarks. Future studies should also consider incorporating ancestry estimates to further validate the reported associations.

## Conclusion

*BMP2* rs1005464 might be associated with variations in the cranial base size. No strong associations were observed between other cranial base shape and symmetry variables and any of the evaluated SNPs.

## Supplementary Information

Supplementary Material 1.

## Data Availability

The datasets used and/or analysed during the current study are available from the corresponding author on reasonable request.

## Abbreviations

BMP: Bone morphogenetic proteins
SMAD6: Mothers Against Decapentaplegic Homolog 6
RUNX2: Runt-related Transcription Factor 2
SNP: Single nucleotide polymorphism
CBCT: Cone beam computed tomography
ICC: Intraclass correlation coefficient
PC: Principal component
