# Supplementary material for: Association between single-nucleotide polymorphisms in endochondral development-related genes and 3D phenotypic variation of the cranial base
Source: Head Face Med. 2026 Jul 21;22:62. doi: 10.1186/s13005-026-00644-8 (PMC13397726; doi:10.1186/s13005-026-00644-8)
Supplement: Supplementary file 1 — Supplementary Material 1. [file 13005_2026_644_MOESM1_ESM.docx]

**Table S1.** 3D landmarks identification method error

| **Landmark** | **Coordinate** | **ICC (95% CI)** | **Dahlberg's Formula** | **Bland-Altman  Bias - Estimate (95% CI)** |
| --- | --- | --- | --- | --- |
| Nasion | X | 0.79 (0.56, 0.91) | 0.39 | -0.11 (-0.35, 0.14) |
|  | Y | 1.00 (0.99, 1.00) | 0.20 | 0.08 (-0.05, 0.20) |
|  | Z | 0.99 (0.97, 1.00) | 0.35 | -0.08 (-0.30, 0.14) |
| Inferior Sella | X | 0.83 (0.65, 0.92) | 0.32 | -0.06 (-0.25, 0.14) |
|  | Y | 0.99 (0.99, 1.00) | 0.25 | -0.05 (-0.20, 0.10) |
|  | Z | 1.00 (1.00, 1.00) | 0.12 | -0.03 (-0.10, 0.04) |
| Foramen Rotundum (right) | X | 1.00 (1.00, 1.00) | 0.10 | 0.02 (-0.04, 0.08) |
|  | Y | 0.99 (0.98, 1.00) | 0.28 | -0.07 (-0.24, 0.11) |
|  | Z | 1.00 (1.00, 1.00) | 0.12 | -0.05 (-0.11, 0.02) |
| Foramen Ovale (right) | X | 0.98 (0.96, 0.99) | 0.27 | 0.04 (-0.13, 0.21) |
|  | Y | 0.99 (0.98, 1.00) | 0.28 | -0.11 (-0.28, 0.05) |
|  | Z | 1.00 (0.99, 1.00) | 0.23 | -0.05 (-0.19, 0.09) |
| Foramen Spinosum (right) | X | 0.95 (0.89, 0.98) | 0.36 | 0.09 (-0.13, 0.31) |
|  | Y | 0.96 (0.90, 0.98) | 0.70 | 0.27 (-0.14, 0.69) |
|  | Z | 0.99 (0.97, 0.99) | 0.44 | 0.07 (-0.20, 0.34) |
| Foramen Rotundum (left) | X | 1.00 (0.99, 1.00) | 0.14 | 0.06 (-0.02, 0.14) |
|  | Y | 0.99 (0.97, 1.00) | 0.39 | 0.03 (-0.21, 0.27) |
|  | Z | 1.00 (0.99, 1.00) | 0.23 | 0.03 (-0.11, 0.17) |
| Foramen Ovale (left) | X | 0.97 0.93, 0.99) | 0.28 | -0.03 (-0.20, 0.13) |
|  | Y | 1.00 (0.99, 1.00) | 0.26 | 0.00 (-0.16, 0.16) |
|  | Z | 0.99 (0.98, 1.00) | 0.33 | 0.05 (-0.15, 0.25) |
| Foramen Spinosum (left) | X | 0.94 (0.86, 0.97) | 0.43 | -0.03 (-0.29, 0.24) |
|  | Y | 0.99 (0.97, 0.99) | 0.46 | -0.06 (-0.34, 0.23) |
|  | Z | 0.99 (0.99, 1.00) | 0.28 | -0.13 (-0.29, 0.04) |
| Basion | X | 0.92 (0.83, 0.97) | 0.32 | 0.06 (-0.14, 0.25) |
|  | Y | 1.00 (0.99, 1.00) | 0.24 | -0.07 (-0.21, 0.07) |
|  | Z | 1.00 (1.00, 1.00) | 0.13 | 0.02 (-0.06, 0.10) |
| Glenoid fossa (right) | X | 0.95 (0.88, 0.98) | 0.58 | 0.13 (-0.22, 0.48) |
|  | Y | 0.98 (0.95, 0.99) | 0.39 | 0.17 (-0.06, 0.40) |
|  | Z | 1.00 (1.00, 1.00) | 0.12 | 0.01 (-0.07, 0.08) |
| Glenoid fossa (left) | X | 0.98 (0.96, 0.99) | 0.31 | -0.07 (-0.25, 0.12) |
|  | Y | 1.00 (0.99, 1.00) | 0.29 | 0.10 (-0.08, 0.27) |
|  | Z | 1.00 (1.00, 1.00) | 0.11 | 0.06 (-0.01, 0.12) |

ICC – Intraclass correlation coefficient, CI – Confidence interval.

**Table S2.** Description of cranial base shape configurations for most negative (-β) or positive (+β) PC scores

| **PCs** | **Symmetric component** | |  | **Asymmetric component** | |
| --- | --- | --- | --- | --- | --- |
|  | **-β** | **+β** |  | **-β** | **+β** |
| PC1 | CB relatively shortened in the anteroposterior dimension, relatively widened transversely, and slightly flexed (frontal and occipital bones configuring a more acute CB angle). | CB relatively elongated in the anteroposterior dimension, relatively narrower transversely, and slightly extended (frontal and occipital bones configuring a more obtuse CB angle). |  | Reduced N - GF separation on the right side, anterior CB oriented to the right, right GF located more anteriorly, and left GF located more posteriorly. | Reduced N - GF separation on the left side, anterior CB oriented to the left, left GF located more anteriorly, and right GF located more posteriorly. |
| PC2 | Flexed CB (frontal, sphenoid and occipital bones configuring a more acute CB angle). Sphenoid bone rotated counterclockwise. | Extended CB (frontal, sphenoid and occipital bones configuring a more obtuse CB angle). Sphenoid bone rotated clockwise. |  | Reduced FR - FO separation on the right side and increased FR - FO separation on the left side; FRs, FOs and FSs located more superiorly on the right side, and more inferiorly on the left side; GF on the left side located more posteriorly and superiorly; GF on the right side located more anteriorly and inferiorly. | Reduced FR - FO separation on the left side and increased FR - FO separation on the right side; FRs, FOs and FSs located more superiorly on the left side, and more inferiorly on the right side; GF on the left side located more anteriorly and inferiorly; GF on the right side located more posteriorly and superiorly. |
| PC3 | Relatively reduced anterior CB configuration, slightly increased posterior CB, increased inter FR separation, FRs located more superiorly, and GFs located more inferiorly and posteriorly. | Relatively increased anterior CB, relatively reduced posterior CB, decreased inter FR separation, FRs located more inferiorly, and GFs located more superiorly and anteriorly. |  | Transverse constriction of the right half of the CB and widening of the left half, right FR located more superiorly, left FR located more inferiorly. | Transverse constriction of the left half of the CB and widening of the right half, left FR located more superiorly, right FR located more inferiorly. |
| PC4 | Relatively reduced anterior CB configuration, markedly increased posterior CB configuration, and GFs located more superiorly. | Relatively increased anterior CB configuration, markedly decreased posterior CB configuration, and GFs located more inferiorly. |  | Reduced Ba - GF separation on the left side, posterior CB oriented to the left, left FR located more inferiorly, right FR located more superiorly. | Reduced Ba - GF separation on the right side, posterior CB oriented to the right, right FR located more inferiorly, left FR located more superiorly. |
| PC5 | Increased inter GF separation; decreased inter FR separation; sphenoid bone showing relative vertical elongation and counterclockwise rotation; GFs, FOs, and FSs located more inferiorly. | Decreased inter GF separation; increased inter FR separation; sphenoid bone showing relative vertical compression and clockwise rotation; GFs, FOs, and FSs located more superiorly. |  | Reduced Ba - GF separation on the right side, posterior CB oriented to the right, left half of the posterior CB widened transversely, FR - FO - FS separation increased on the right side and decreased on the left side, left FR located more superiorly, left FO and FS located more inferiorly, right FR located more inferiorly, right FO and FS located more superiorly, left GF located more superiorly, and right GF located more inferiorly. | Reduced Ba - GF separation on the left side, posterior CB oriented to the left, right half of the posterior CB widened transversely, FR - FO - FS separation increased on the left side and decreased on the right side, left FR located more inferiorly, left FO and FS located more superiorly, right FR located more superiorly, right FO and FS located more inferiorly, left GF located more inferiorly, and right GF located more superiorly. |
| PC6 | Markedly decreased anterior CB configuration, increased inter GF separation; decreased inter FR separation; sphenoid bone showing relative anteroposterior elongation. | Markedly increased anterior CB configuration, decreased inter GF separation; increased inter FR separation; sphenoid bone showing slight relative anteroposterior reduction together with counterclockwise rotation. |  | Relative transverse constriction of the left half of the CB and transverse expansion of the right half of the CB, FR - FO - FS separation increased on the right side and decreased on the left side, left FR located more inferiorly, right FR located more superiorly, left FS located more laterally, right GF located more anteriorly, and left GF located more posteriorly. | Relative transverse constriction of the right half of the CB and transverse expansion of the left half of the CB, FR - FO - FS separation increased on the left side and decreased on the left side, left FR located more superiorly, right FR located more inferiorly, right FS located more laterally, right GF located more posteriorly, and left GF located more anteriorly. |
| PC7 | Relatively decreased anterior CB configuration, markedly increased posterior CB configuration, slightly extended CB, sphenoid bone rotated clockwise, and GFs located more anteriorly and inferiorly. | Relatively increased anterior CB configuration, markedly decreased posterior CB configuration, slightly flexed CB, sphenoid bone rotated counterclockwise, and GFs located more posteriorly and superiorly. |  | Reduced Ba - GF separation on the left side, posterior CB oriented to the left, relative transverse constriction of the left half of the CB and transverse expansion of the right half of the CB, left FR located more superiorly, left FO and FS located more inferiorly, left FS located more laterally, right FR located more inferiorly, right FO and FS located more superiorly. | Reduced Ba - GF separation on the right side, posterior CB oriented to the right, relative transverse constriction of the right half of the CB and transverse expansion of the left half of the CB, right FR located more superiorly, right FO and FS located more inferiorly, right FS located more laterally, left FR located more inferiorly, left FO and FS located more superiorly. |

**Table S3.** Effect of single nucleotide polymorphisms studied on cranial base phenotypes

| **Cranial phenotype** | **Gene SNP (1/2)**† | **Genotypes** | **Model coefficients** | | | |  | **Model fit measures** | |
| --- | --- | --- | --- | --- | --- | --- | --- | --- | --- |
|  |  |  | **β** | **95% CI** | | ***P* value** |  | **F test**  ***P* value** | **Adjusted R^2^** |
|  |  |  |  | **lower** | **upper** |  |  |  |  |
| PC1 - Symmetric component | *BMP2* rs1005464 (A/G) | AG vs. GG | 0.002 | -0.010 | 0.014 | 0.756 |  | 0.356 | 0.004 |
|  |  | AA vs. GG | 0.004 | -0.021 | 0.028 | 0.768 |  |  |  |
|  |  | AG+AA vs. GG | 0.002 | -0.009 | 0.014 | 0.710 |  | 0.222 | 0.014 |
|  |  | AA vs. AG+GG | 0.003 | -0.021 | 0.027 | 0.806 |  | 0.230 | 0.013 |
|  | *BMP2* rs235768 (A/T) | AT vs. TT | -0.004 | -0.015 | 0.008 | 0.525 |  | 0.319 | 0.007 |
|  |  | AA vs. TT | 0.000 | -0.021 | 0.022 | 0.951 |  |  |  |
|  |  | AT+AA vs. TT | -0.003 | -0.014 | 0.008 | 0.582 |  | 0.207 | 0.016 |
|  |  | AA vs. AT+TT | 0.003 | -0.018 | 0.023 | 0.806 |  | 0.230 | 0.013 |
|  | *BMP4* rs17563 (G/A) | AG vs. AA | 0.000 | -0.014 | 0.012 | 0.899 |  | 0.297 | 0.009 |
|  |  | GG vs. AA | -0.007 | -0.025 | 0.011 | 0.443 |  |  |  |
|  |  | AG+GG vs. AA | -0.002 | -0.014 | 0.010 | 0.752 |  | 0.226 | 0.014 |
|  |  | GG vs. AG+AA | -0.007 | -0.022 | 0.009 | 0.417 |  | 0.178 | 0.019 |
|  | *RUNX2* rs59983488 (T/G) | GT vs. GG | 0.000 | -0.012 | 0.012 | 0.978 |  | 0.290 | 0.010 |
|  |  | TT vs. GG | -0.014 | -0.048 | 0.019 | 0.398 |  |  |  |
|  |  | GT+TT vs. GG | 0.000 | -0.012 | 0.011 | 0.874 |  | 0.233 | 0.013 |
|  |  | TT vs. GT+GG | -0.014 | -0.048 | 0.019 | 0.390 |  | 0.172 | 0.020 |
|  | *RUNX2* rs1200425 (A/G) | AG vs. GG | -0.005 | -0.017 | 0.008 | 0.467 |  | 0.121 | 0.033 |
|  |  | AA vs. GG | 0.009 | -0.007 | 0.026 | 0.277 |  |  |  |
|  |  | AG+AA vs. GG | -0.001 | -0.013 | 0.011 | 0.850 |  | 0.232 | 0.013 |
|  |  | AA vs. AG+GG | 0.012 | -0.003 | 0.027 | 0.112 |  | 0.079 | 0.037 |
|  | *SMAD6* rs2119261 (T/C) | CT vs. CC | -0.003 | -0.015 | 0.010 | 0.663 |  | 0.235 | 0.016 |
|  |  | TT vs. CC | 0.007 | -0.010 | 0.024 | 0.443 |  |  |  |
|  |  | CT+TT vs. CC | 0.000 | -0.012 | 0.011 | 0.928 |  | 0.235 | 0.013 |
|  |  | TT vs. CT+CC | 0.008 | -0.007 | 0.023 | 0.288 |  | 0.145 | 0.024 |
|  | *SMAD6* rs3934908 (T/C) | CT vs. CC | 0.000 | -0.013 | 0.012 | 0.956 |  | 0.100 | 0.037 |
|  |  | TT vs. CC | -0.014 | -0.030 | 0.003 | 0.101 |  |  |  |
|  |  | CT+TT vs. CC | -0.004 | -0.016 | 0.008 | 0.540 |  | 0.201 | 0.016 |
|  |  | TT vs. CT+CC | -0.013 | -0.027 | 0.000 | 0.061 |  | 0.050* | 0.047 |
| PC2 - Symmetric component | *BMP2* rs1005464 (A/G) | AG vs. GG | -0.004 | -0.014 | 0.005 | 0.373 |  | 0.369 | 0.003 |
|  |  | AA vs. GG | -0.016 | -0.035 | 0.003 | 0.106 |  |  |  |
|  |  | AG+AA vs. GG | -0.006 | -0.015 | 0.003 | 0.194 |  | 0.396 | 0.000 |
|  |  | AA vs. AG+GG | -0.014 | -0.033 | 0.005 | 0.137 |  | 0.322 | 0.005 |
|  | *BMP2* rs235768 (A/T) | AT vs. TT | -0.003 | -0.012 | 0.006 | 0.522 |  | 0.795 | -0.023 |
|  |  | AA vs. TT | -0.002 | -0.019 | 0.015 | 0.810 |  |  |  |
|  |  | AT+AA vs. TT | -0.003 | -0.012 | 0.006 | 0.523 |  | 0.642 | -0.013 |
|  |  | AA vs. AT+TT | 0.000 | -0.017 | 0.016 | 0.946 |  | 0.736 | -0.017 |
|  | *BMP4* rs17563 (G/A) | AG vs. AA | -0.009 | -0.019 | 0.000 | 0.072 |  | 0.122 | 0.032 |
|  |  | GG vs. AA | -0.017 | -0.031 | -0.003 | 0.020* |  |  |  |
|  |  | AG+GG vs. AA | -0.011 | -0.020 | 0.000 | 0.033* |  | 0.118 | 0.028 |
|  |  | GG vs. AG+AA | -0.011 | -0.023 | 0.002 | 0.099 |  | 0.260 | 0.010 |
|  | *RUNX2* rs59983488 (T/G) | GT vs. GG | 0.004 | -0.006 | 0.013 | 0.458 |  | 0.765 | -0.021 |
|  |  | TT vs. GG | 0.000 | -0.028 | 0.026 | 0.953 |  |  |  |
|  |  | GT+TT vs. GG | 0.003 | -0.006 | 0.012 | 0.488 |  | 0.626 | -0.012 |
|  |  | TT vs. GT+GG | -0.002 | -0.029 | 0.025 | 0.874 |  | 0.731 | -0.017 |
|  | *RUNX2* rs1200425 (A/G) | AG vs. GG | -0.007 | -0.017 | 0.003 | 0.177 |  | 0.105 | 0.036 |
|  |  | AA vs. GG | 0.009 | -0.004 | 0.022 | 0.190 |  |  |  |
|  |  | AG+AA vs. GG | -0.003 | -0.012 | 0.007 | 0.554 |  | 0.655 | -0.013 |
|  |  | AA vs. AG+GG | 0.013 | 0.001 | 0.025 | 0.033* |  | 0.119 | 0.028 |
|  | *SMAD6* rs2119261 (T/C) | CT vs. CC | 0.004 | -0.006 | 0.014 | 0.444 |  | 0.763 | -0.021 |
|  |  | TT vs. CC | 0.002 | -0.011 | 0.016 | 0.720 |  |  |  |
|  |  | CT+TT vs. CC | 0.004 | -0.006 | 0.013 | 0.458 |  | 0.610 | -0.011 |
|  |  | TT vs. CT+CC | 0.000 | -0.012 | 0.013 | 0.975 |  | 0.737 | -0.017 |
|  | *SMAD6* rs3934908 (T/C) | CT vs. CC | -0.007 | -0.018 | 0.003 | 0.161 |  | 0.390 | 0.002 |
|  |  | TT vs. CC | 0.000 | -0.013 | 0.014 | 0.930 |  |  |  |
|  |  | CT+TT vs. CC | -0.005 | -0.015 | 0.005 | 0.292 |  | 0.496 | -0.006 |
|  |  | TT vs. CT+CC | 0.005 | -0.006 | 0.017 | 0.353 |  | 0.545 | -0.008 |
| PC3 - Symmetric component | *BMP2* rs1005464 (A/G) | AG vs. GG | -0.003 | -0.011 | 0.006 | 0.564 |  | 0.209 | 0.019 |
|  |  | AA vs. GG | -0.003 | -0.021 | 0.014 | 0.726 |  |  |  |
|  |  | AG+AA vs. GG | -0.003 | -0.011 | 0.006 | 0.529 |  | 0.117 | 0.028 |
|  |  | AA vs. AG+GG | -0.002 | -0.019 | 0.015 | 0.801 |  | 0.135 | 0.025 |
|  | *BMP2* rs235768 (A/T) | AT vs. TT | 0.000 | -0.009 | 0.008 | 0.831 |  | 0.190 | 0.021 |
|  |  | AA vs. TT | -0.006 | -0.022 | 0.009 | 0.421 |  |  |  |
|  |  | AT+AA vs. TT | -0.002 | -0.010 | 0.006 | 0.680 |  | 0.129 | 0.026 |
|  |  | AA vs. AT+TT | -0.006 | -0.021 | 0.009 | 0.436 |  | 0.107 | 0.030 |
|  | *BMP4* rs17563 (G/A) | AG vs. AA | 0.012 | 0.003 | 0.021 | 0.012* |  | 0.017* | 0.077 |
|  |  | GG vs. AA | 0.006 | -0.006 | 0.019 | 0.322 |  |  |  |
|  |  | AG+GG vs. AA | 0.011 | 0.002 | 0.019 | 0.017* |  | 0.010* | 0.078 |
|  |  | GG vs. AG+AA | -0.002 | -0.013 | 0.010 | 0.778 |  | 0.134 | 0.025 |
|  | *RUNX2* rs59983488 (T/G) | GT vs. GG | 0.006 | -0.003 | 0.014 | 0.171 |  | 0.093 | 0.039 |
|  |  | TT vs. GG | -0.007 | -0.031 | 0.017 | 0.558 |  |  |  |
|  |  | GT+TT vs. GG | 0.005 | -0.003 | 0.013 | 0.241 |  | 0.076 | 0.038 |
|  |  | TT vs. GT+GG | -0.009 | -0.033 | 0.015 | 0.442 |  | 0.107 | 0.030 |
|  | *RUNX2* rs1200425 (A/G) | AG vs. GG | 0.000 | -0.010 | 0.008 | 0.840 |  | 0.160 | 0.026 |
|  |  | AA vs. GG | 0.005 | -0.007 | 0.017 | 0.410 |  |  |  |
|  |  | AG+AA vs. GG | 0.000 | -0.008 | 0.009 | 0.896 |  | 0.138 | 0.025 |
|  |  | AA vs. AG+GG | 0.006 | -0.005 | 0.016 | 0.303 |  | 0.087 | 0.035 |
|  | *SMAD6* rs2119261 (T/C) | CT vs. CC | 0.003 | -0.006 | 0.012 | 0.456 |  | 0.193 | 0.021 |
|  |  | TT vs. CC | 0.000 | -0.012 | 0.013 | 0.909 |  |  |  |
|  |  | CT+TT vs. CC | 0.003 | -0.006 | 0.011 | 0.521 |  | 0.116 | 0.029 |
|  |  | TT vs. CT+CC | -0.001 | -0.012 | 0.010 | 0.818 |  | 0.136 | 0.025 |
|  | *SMAD6* rs3934908 (T/C) | CT vs. CC | -0.005 | -0.014 | 0.004 | 0.297 |  | 0.120 | 0.033 |
|  |  | TT vs. CC | -0.008 | -0.020 | 0.004 | 0.205 |  |  |  |
|  |  | CT+TT vs. CC | -0.006 | -0.015 | 0.003 | 0.209 |  | 0.069 | 0.040 |
|  |  | TT vs. CT+CC | -0.004 | -0.015 | 0.006 | 0.391 |  | 0.101 | 0.032 |
| PC4 - Symmetric component | *BMP2* rs1005464 (A/G) | AG vs. GG | -0.007 | -0.014 | 0.000 | 0.065 |  | 0.002* | 0.125 |
|  |  | AA vs. GG | 0.002 | -0.013 | 0.017 | 0.782 |  |  |  |
|  |  | AG+AA vs. GG | -0.006 | -0.012 | 0.001 | 0.116 |  | 0.001* | 0.122 |
|  |  | AA vs. AG+GG | 0.005 | -0.010 | 0.019 | 0.543 |  | 0.003* | 0.103 |
|  | *BMP2* rs235768 (A/T) | AT vs. TT | 0.008 | 0.000 | 0.015 | 0.029* |  | <0.001* | 0.135 |
|  |  | AA vs. TT | 0.006 | -0.007 | 0.019 | 0.339 |  |  |  |
|  |  | AT+AA vs. TT | 0.008 | 0.000 | 0.014 | 0.027* |  | <0.001* | 0.143 |
|  |  | AA vs. AT+TT | 0.002 | -0.010 | 0.015 | 0.719 |  | 0.003* | 0.101 |
|  | *BMP4* rs17563 (G/A) | AG vs. AA | 0.003 | -0.005 | 0.011 | 0.453 |  | 0.003* | 0.112 |
|  |  | GG vs. AA | -0.005 | -0.016 | 0.007 | 0.413 |  |  |  |
|  |  | AG+GG vs. AA | 0.002 | -0.006 | 0.009 | 0.686 |  | 0.003* | 0.101 |
|  |  | GG vs. AG+AA | -0.007 | -0.016 | 0.003 | 0.176 |  | 0.001* | 0.116 |
|  | *RUNX2* rs59983488 (T/G) | GT vs. GG | 0.003 | -0.005 | 0.010 | 0.492 |  | 0.006* | 0.098 |
|  |  | TT vs. GG | -0.005 | -0.026 | 0.016 | 0.635 |  |  |  |
|  |  | GT+TT vs. GG | 0.002 | -0.005 | 0.009 | 0.582 |  | 0.003* | 0.103 |
|  |  | TT vs. GT+GG | -0.006 | -0.027 | 0.015 | 0.568 |  | 0.003* | 0.103 |
|  | *RUNX2* rs1200425 (A/G) | AG vs. GG | 0.003 | -0.005 | 0.010 | 0.482 |  | 0.003* | 0.110 |
|  |  | AA vs. GG | -0.004 | -0.015 | 0.006 | 0.412 |  |  |  |
|  |  | AG+AA vs. GG | 0.000 | -0.006 | 0.008 | 0.791 |  | 0.003* | 0.100 |
|  |  | AA vs. AG+GG | -0.006 | -0.015 | 0.003 | 0.201 |  | 0.002* | 0.114 |
|  | *SMAD6* rs2119261 (T/C) | CT vs. CC | 0.000 | -0.007 | 0.008 | 0.912 |  | 0.004* | 0.107 |
|  |  | TT vs. CC | -0.006 | -0.017 | 0.004 | 0.249 |  |  |  |
|  |  | CT+TT vs. CC | -0.001 | -0.008 | 0.006 | 0.762 |  | 0.003* | 0.101 |
|  |  | TT vs. CT+CC | -0.006 | -0.016 | 0.003 | 0.182 |  | 0.001* | 0.116 |
|  | *SMAD6* rs3934908 (T/C) | CT vs. CC | -0.002 | -0.010 | 0.006 | 0.587 |  | 0.003* | 0.113 |
|  |  | TT vs. CC | 0.005 | -0.005 | 0.015 | 0.319 |  |  |  |
|  |  | CT+TT vs. CC | 0.000 | -0.008 | 0.007 | 0.943 |  | 0.003* | 0.100 |
|  |  | TT vs. CT+CC | 0.007 | -0.002 | 0.015 | 0.139 |  | 0.001* | 0.119 |
| PC5 - Symmetric component | *BMP2* rs1005464 (A/G) | AG vs. GG | 0.003 | -0.004 | 0.011 | 0.377 |  | 0.600 | -0.012 |
|  |  | AA vs. GG | 0.001 | -0.013 | 0.016 | 0.860 |  |  |  |
|  |  | AG+AA vs. GG | 0.003 | -0.004 | 0.010 | 0.394 |  | 0.440 | -0.003 |
|  |  | AA vs. AG+GG | 0.000 | -0.014 | 0.015 | 0.984 |  | 0.578 | -0.010 |
|  | *BMP2* rs235768 (A/T) | AT vs. TT | -0.006 | -0.013 | 0.001 | 0.115 |  | 0.340 | 0.006 |
|  |  | AA vs. TT | -0.002 | -0.015 | 0.011 | 0.802 |  |  |  |
|  |  | AT+AA vs. TT | -0.005 | -0.012 | 0.002 | 0.140 |  | 0.244 | 0.012 |
|  |  | AA vs. AT+TT | 0.001 | -0.011 | 0.014 | 0.850 |  | 0.571 | -0.010 |
|  | *BMP4* rs17563 (G/A) | AG vs. AA | 0.002 | -0.004 | 0.010 | 0.529 |  | 0.526 | -0.008 |
|  |  | GG vs. AA | -0.003 | -0.014 | 0.008 | 0.625 |  |  |  |
|  |  | AG+GG vs. AA | 0.002 | -0.006 | 0.009 | 0.693 |  | 0.546 | -0.008 |
|  |  | GG vs. AG+AA | -0.004 | -0.014 | 0.005 | 0.363 |  | 0.422 | -0.002 |
|  | *RUNX2* rs59983488 (T/G) | GT vs. GG | -0.002 | -0.010 | 0.005 | 0.511 |  | 0.655 | -0.015 |
|  |  | TT vs. GG | 0.001 | -0.019 | 0.022 | 0.909 |  |  |  |
|  |  | GT+TT vs. GG | -0.002 | -0.009 | 0.005 | 0.547 |  | 0.506 | -0.006 |
|  |  | TT vs. GT+GG | 0.002 | -0.018 | 0.022 | 0.839 |  | 0.570 | -0.009 |
|  | *RUNX2* rs1200425 (A/G) | AG vs. GG | 0.002 | -0.006 | 0.009 | 0.622 |  | 0.477 | -0.005 |
|  |  | AA vs. GG | 0.006 | -0.004 | 0.017 | 0.218 |  |  |  |
|  |  | AG+AA vs. GG | 0.003 | -0.004 | 0.010 | 0.410 |  | 0.448 | -0.003 |
|  |  | AA vs. AG+GG | 0.005 | -0.004 | 0.014 | 0.256 |  | 0.351 | 0.003 |
|  | *SMAD6* rs2119261 (T/C) | CT vs. CC | 0.007 | 0.000 | 0.014 | 0.071 |  | 0.235 | 0.016 |
|  |  | TT vs. CC | 0.007 | -0.004 | 0.017 | 0.206 |  |  |  |
|  |  | CT+TT vs. CC | 0.007 | 0.000 | 0.014 | 0.059 |  | 0.134 | 0.025 |
|  |  | TT vs. CT+CC | 0.003 | -0.007 | 0.012 | 0.595 |  | 0.521 | -0.007 |
|  | *SMAD6* rs3934908 (T/C) | CT vs. CC | 0.005 | -0.003 | 0.013 | 0.198 |  | 0.456 | -0.003 |
|  |  | TT vs. CC | 0.004 | -0.006 | 0.014 | 0.479 |  |  |  |
|  |  | CT+TT vs. CC | 0.005 | -0.003 | 0.012 | 0.211 |  | 0.314 | 0.006 |
|  |  | TT vs. CT+CC | 0.000 | -0.008 | 0.009 | 0.953 |  | 0.578 | -0.010 |
| PC6 - Symmetric component | *BMP2* rs1005464 (A/G) | AG vs. GG | 0.003 | -0.004 | 0.009 | 0.421 |  | 0.002* | 0.123 |
|  |  | AA vs. GG | -0.007 | -0.020 | 0.006 | 0.300 |  |  |  |
|  |  | AG+AA vs. GG | 0.001 | -0.005 | 0.007 | 0.691 |  | 0.002* | 0.115 |
|  |  | AA vs. AG+GG | -0.008 | -0.021 | 0.005 | 0.231 |  | <0.001* | 0.126 |
|  | *BMP2* rs235768 (A/T) | AT vs. TT | 0.001 | -0.005 | 0.007 | 0.749 |  | <0.001* | 0.140 |
|  |  | AA vs. TT | -0.012 | -0.022 | 0.000 | 0.070 |  |  |  |
|  |  | AT+AA vs. TT | 0.000 | -0.007 | 0.005 | 0.830 |  | 0.002* | 0.114 |
|  |  | AA vs. AT+TT | -0.011 | -0.022 | 0.000 | 0.048* |  | <0.001* | 0.147 |
|  | *BMP4* rs17563 (G/A) | AG vs. AA | 0.001 | -0.005 | 0.008 | 0.670 |  | 0.003* | 0.116 |
|  |  | GG vs. AA | 0.005 | -0.004 | 0.015 | 0.272 |  |  |  |
|  |  | AG+GG vs. AA | 0.002 | -0.004 | 0.009 | 0.510 |  | 0.001* | 0.117 |
|  |  | GG vs. AG+AA | 0.004 | -0.004 | 0.013 | 0.305 |  | 0.001* | 0.123 |
|  | *RUNX2* rs59983488 (T/G) | GT vs. GG | 0.000 | -0.006 | 0.007 | 0.973 |  | 0.004* | 0.108 |
|  |  | TT vs. GG | 0.006 | -0.012 | 0.024 | 0.520 |  |  |  |
|  |  | GT+TT vs. GG | 0.000 | -0.006 | 0.007 | 0.863 |  | 0.002* | 0.114 |
|  |  | TT vs. GT+GG | 0.006 | -0.012 | 0.024 | 0.517 |  | 0.001* | 0.117 |
|  | *RUNX2* rs1200425 (A/G) | AG vs. GG | 0.003 | -0.004 | 0.010 | 0.423 |  | 0.003* | 0.113 |
|  |  | AA vs. GG | 0.000 | -0.010 | 0.009 | 0.922 |  |  |  |
|  |  | AG+AA vs. GG | 0.002 | -0.004 | 0.008 | 0.548 |  | 0.001* | 0.117 |
|  |  | AA vs. AG+GG | -0.002 | -0.010 | 0.006 | 0.604 |  | 0.001* | 0.116 |
|  | *SMAD6* rs2119261 (T/C) | CT vs. CC | 0.000 | -0.008 | 0.006 | 0.770 |  | 0.004* | 0.105 |
|  |  | TT vs. CC | 0.000 | -0.010 | 0.009 | 0.952 |  |  |  |
|  |  | CT+TT vs. CC | 0.000 | -0.007 | 0.006 | 0.797 |  | 0.002* | 0.114 |
|  |  | TT vs. CT+CC | 0.000 | -0.008 | 0.009 | 0.942 |  | 0.002* | 0.114 |
|  | *SMAD6* rs3934908 (T/C) | CT vs. CC | 0.000 | -0.006 | 0.008 | 0.818 |  | 0.002* | 0.124 |
|  |  | TT vs. CC | -0.005 | -0.014 | 0.004 | 0.249 |  |  |  |
|  |  | CT+TT vs. CC | 0.000 | -0.007 | 0.006 | 0.821 |  | 0.002* | 0.114 |
|  |  | TT vs. CT+CC | -0.006 | -0.013 | 0.002 | 0.139 |  | <0.001* | 0.133 |
| PC7 - Symmetric component | *BMP2* rs1005464 (A/G) | AG vs. GG | 0.000 | -0.006 | 0.005 | 0.848 |  | 0.130 | 0.031 |
|  |  | AA vs. GG | -0.015 | -0.027 | -0.004 | 0.009* |  |  |  |
|  |  | AG+AA vs. GG | -0.003 | -0.008 | 0.003 | 0.328 |  | 0.805 | -0.020 |
|  |  | AA vs. AG+GG | -0.015 | -0.026 | -0.004 | 0.008* |  | 0.068 | 0.040 |
|  | *BMP2* rs235768 (A/T) | AT vs. TT | 0.002 | -0.004 | 0.008 | 0.483 |  | 0.970 | -0.035 |
|  |  | AA vs. TT | 0.002 | -0.009 | 0.012 | 0.759 |  |  |  |
|  |  | AT+AA vs. TT | 0.002 | -0.003 | 0.007 | 0.477 |  | 0.913 | -0.024 |
|  |  | AA vs. AT+TT | 0.000 | -0.009 | 0.011 | 0.905 |  | 0.999 | -0.029 |
|  | *BMP4* rs17563 (G/A) | AG vs. AA | 0.000 | -0.006 | 0.007 | 0.828 |  | 0.997 | -0.038 |
|  |  | GG vs. AA | 0.002 | -0.007 | 0.011 | 0.715 |  |  |  |
|  |  | AG+GG vs. AA | 0.000 | -0.005 | 0.007 | 0.777 |  | 0.992 | -0.029 |
|  |  | GG vs. AG+AA | 0.001 | -0.007 | 0.009 | 0.765 |  | 0.991 | -0.029 |
|  | *RUNX2* rs59983488 (T/G) | GT vs. GG | -0.005 | -0.011 | 0.000 | 0.074 |  | 0.515 | -0.007 |
|  |  | TT vs. GG | -0.002 | -0.018 | 0.014 | 0.807 |  |  |  |
|  |  | GT+TT vs. GG | -0.005 | -0.010 | 0.000 | 0.079 |  | 0.372 | 0.002 |
|  |  | TT vs. GT+GG | 0.000 | -0.016 | 0.016 | 0.994 |  | 0.999 | -0.030 |
|  | *RUNX2* rs1200425 (A/G) | AG vs. GG | 0.004 | -0.002 | 0.010 | 0.178 |  | 0.144 | 0.028 |
|  |  | AA vs. GG | 0.011 | 0.003 | 0.018 | 0.010* |  |  |  |
|  |  | AG+AA vs. GG | 0.006 | 0.000 | 0.011 | 0.050 |  | 0.274 | 0.009 |
|  |  | AA vs. AG+GG | 0.008 | 0.000 | 0.015 | 0.026* |  | 0.170 | 0.020 |
|  | *SMAD6* rs2119261 (T/C) | CT vs. CC | 0.001 | -0.005 | 0.007 | 0.698 |  | 0.944 | -0.032 |
|  |  | TT vs. CC | -0.002 | -0.010 | 0.006 | 0.604 |  |  |  |
|  |  | CT+TT vs. CC | 0.000 | -0.005 | 0.006 | 0.891 |  | 0.998 | -0.029 |
|  |  | TT vs. CT+CC | -0.003 | -0.010 | 0.005 | 0.446 |  | 0.895 | -0.024 |
|  | *SMAD6* rs3934908 (T/C) | CT vs. CC | -0.002 | -0.009 | 0.004 | 0.472 |  | 0.903 | -0.029 |
|  |  | TT vs. CC | -0.004 | -0.012 | 0.004 | 0.331 |  |  |  |
|  |  | CT+TT vs. CC | -0.003 | -0.009 | 0.003 | 0.368 |  | 0.841 | -0.021 |
|  |  | TT vs. CT+CC | -0.002 | -0.009 | 0.004 | 0.479 |  | 0.914 | -0.024 |
| PC1 - Asymmetric component | *BMP2* rs1005464 (A/G) | AG vs. GG | 0.004 | -0.001 | 0.009 | 0.164 |  | 0.286 | 0.010 |
|  |  | AA vs. GG | 0.008 | -0.002 | 0.018 | 0.113 |  |  |  |
|  |  | AG+AA vs. GG | 0.004 | 0.000 | 0.009 | 0.082 |  | 0.235 | 0.013 |
|  |  | AA vs. AG+GG | 0.007 | -0.003 | 0.017 | 0.175 |  | 0.383 | 0.000 |
|  | *BMP2* rs235768 (A/T) | AT vs. TT | 0.001 | -0.004 | 0.006 | 0.587 |  | 0.340 | 0.006 |
|  |  | AA vs. TT | -0.007 | -0.016 | 0.002 | 0.128 |  |  |  |
|  |  | AT+AA vs. TT | 0.000 | -0.005 | 0.005 | 0.951 |  | 0.752 | -0.018 |
|  |  | AA vs. AT+TT | -0.008 | -0.017 | 0.001 | 0.082 |  | 0.236 | 0.013 |
|  | *BMP4* rs17563 (G/A) | AG vs. AA | 0.000 | -0.006 | 0.005 | 0.788 |  | 0.549 | -0.009 |
|  |  | GG vs. AA | -0.005 | -0.013 | 0.003 | 0.197 |  |  |  |
|  |  | AG+GG vs. AA | -0.002 | -0.007 | 0.004 | 0.564 |  | 0.674 | -0.014 |
|  |  | GG vs. AG+AA | -0.005 | -0.011 | 0.002 | 0.183 |  | 0.393 | 0.000 |
|  | *RUNX2* rs59983488 (T/G) | GT vs. GG | 0.001 | -0.004 | 0.006 | 0.599 |  | 0.393 | 0.001 |
|  |  | TT vs. GG | 0.012 | -0.002 | 0.026 | 0.096 |  |  |  |
|  |  | GT+TT vs. GG | 0.002 | -0.003 | 0.007 | 0.392 |  | 0.585 | -0.010 |
|  |  | TT vs. GT+GG | 0.012 | -0.003 | 0.026 | 0.106 |  | 0.280 | 0.008 |
|  | *RUNX2* rs1200425 (A/G) | AG vs. GG | 0.003 | -0.003 | 0.008 | 0.293 |  | 0.676 | -0.016 |
|  |  | AA vs. GG | 0.002 | -0.005 | 0.009 | 0.567 |  |  |  |
|  |  | AG+AA vs. GG | 0.003 | -0.002 | 0.008 | 0.300 |  | 0.516 | -0.007 |
|  |  | AA vs. AG+GG | 0.000 | -0.006 | 0.007 | 0.915 |  | 0.750 | -0.018 |
|  | *SMAD6* rs2119261 (T/C) | CT vs. CC | 0.005 | 0.000 | 0.010 | 0.068 |  | 0.292 | 0.010 |
|  |  | TT vs. CC | 0.005 | -0.002 | 0.012 | 0.174 |  |  |  |
|  |  | CT+TT vs. CC | 0.005 | 0.000 | 0.010 | 0.053 |  | 0.173 | 0.020 |
|  |  | TT vs. CT+CC | 0.002 | -0.004 | 0.009 | 0.529 |  | 0.660 | -0.014 |
|  | *SMAD6* rs3934908 (T/C) | CT vs. CC | 0.003 | -0.003 | 0.008 | 0.288 |  | 0.320 | 0.007 |
|  |  | TT vs. CC | -0.003 | -0.010 | 0.004 | 0.434 |  |  |  |
|  |  | CT+TT vs. CC | 0.001 | -0.004 | 0.007 | 0.586 |  | 0.682 | -0.015 |
|  |  | TT vs. CT+CC | -0.005 | -0.011 | 0.001 | 0.126 |  | 0.312 | 0.006 |
| PC2 - Asymmetric component | *BMP2* rs1005464 (A/G) | AG vs. GG | 0.000 | -0.004 | 0.004 | 0.967 |  | 0.422 | 0.000 |
|  |  | AA vs. GG | -0.004 | -0.012 | 0.004 | 0.321 |  |  |  |
|  |  | AG+AA vs. GG | 0.000 | -0.004 | 0.003 | 0.785 |  | 0.404 | 0.000 |
|  |  | AA vs. AG+GG | -0.004 | -0.011 | 0.004 | 0.308 |  | 0.273 | 0.009 |
|  | *BMP2* rs235768 (A/T) | AT vs. TT | 0.001 | -0.002 | 0.005 | 0.516 |  | 0.353 | 0.005 |
|  |  | AA vs. TT | -0.003 | -0.010 | 0.004 | 0.394 |  |  |  |
|  |  | AT+AA vs. TT | 0.000 | -0.003 | 0.004 | 0.732 |  | 0.397 | 0.000 |
|  |  | AA vs. AT+TT | -0.004 | -0.010 | 0.003 | 0.284 |  | 0.261 | 0.010 |
|  | *BMP4* rs17563 (G/A) | AG vs. AA | 0.001 | -0.003 | 0.005 | 0.496 |  | 0.494 | -0.006 |
|  |  | GG vs. AA | 0.000 | -0.006 | 0.006 | 0.945 |  |  |  |
|  |  | AG+GG vs. AA | 0.001 | -0.003 | 0.005 | 0.553 |  | 0.362 | 0.002 |
|  |  | GG vs. AG+AA | 0.000 | -0.006 | 0.004 | 0.763 |  | 0.401 | 0.000 |
|  | *RUNX2* rs59983488 (T/G) | GT vs. GG | -0.002 | -0.006 | 0.002 | 0.299 |  | 0.415 | 0.000 |
|  |  | TT vs. GG | -0.001 | -0.012 | 0.010 | 0.845 |  |  |  |
|  |  | GT+TT vs. GG | -0.002 | -0.006 | 0.002 | 0.302 |  | 0.270 | 0.009 |
|  |  | TT vs. GT+GG | 0.000 | -0.011 | 0.010 | 0.953 |  | 0.415 | -0.001 |
|  | *RUNX2* rs1200425 (A/G) | AG vs. GG | -0.002 | -0.006 | 0.002 | 0.329 |  | 0.315 | 0.008 |
|  |  | AA vs. GG | 0.001 | -0.004 | 0.006 | 0.674 |  |  |  |
|  |  | AG+AA vs. GG | -0.001 | -0.005 | 0.003 | 0.534 |  | 0.356 | 0.003 |
|  |  | AA vs. AG+GG | 0.002 | -0.002 | 0.007 | 0.334 |  | 0.285 | 0.008 |
|  | *SMAD6* rs2119261 (T/C) | CT vs. CC | 0.000 | -0.003 | 0.004 | 0.785 |  | 0.572 | -0.010 |
|  |  | TT vs. CC | 0.000 | -0.005 | 0.006 | 0.966 |  |  |  |
|  |  | CT+TT vs. CC | 0.000 | -0.003 | 0.004 | 0.814 |  | 0.407 | 0.000 |
|  |  | TT vs. CT+CC | 0.000 | -0.005 | 0.005 | 0.933 |  | 0.415 | -0.001 |
|  | *SMAD6* rs3934908 (T/C) | CT vs. CC | 0.001 | -0.003 | 0.005 | 0.583 |  | 0.505 | -0.006 |
|  |  | TT vs. CC | 0.002 | -0.004 | 0.007 | 0.518 |  |  |  |
|  |  | CT+TT vs. CC | 0.001 | -0.003 | 0.005 | 0.513 |  | 0.350 | 0.003 |
|  |  | TT vs. CT+CC | 0.000 | -0.004 | 0.006 | 0.669 |  | 0.387 | 0.000 |
| PC3 - Asymmetric component | *BMP2* rs1005464 (A/G) | AG vs. GG | 0.000 | -0.003 | 0.004 | 0.775 |  | 0.925 | -0.031 |
|  |  | AA vs. GG | 0.000 | -0.007 | 0.007 | 0.935 |  |  |  |
|  |  | AG+AA vs. GG | 0.000 | -0.003 | 0.004 | 0.816 |  | 0.836 | -0.021 |
|  |  | AA vs. AG+GG | 0.000 | -0.007 | 0.006 | 0.892 |  | 0.845 | -0.021 |
|  | *BMP2* rs235768 (A/T) | AT vs. TT | 0.000 | -0.003 | 0.004 | 0.953 |  | 0.929 | -0.031 |
|  |  | AA vs. TT | 0.000 | -0.007 | 0.006 | 0.820 |  |  |  |
|  |  | AT+AA vs. TT | 0.000 | -0.003 | 0.003 | 0.992 |  | 0.849 | -0.022 |
|  |  | AA vs. AT+TT | 0.000 | -0.007 | 0.005 | 0.799 |  | 0.834 | -0.021 |
|  | *BMP4* rs17563 (G/A) | AG vs. AA | 0.000 | -0.004 | 0.004 | 0.985 |  | 0.898 | -0.029 |
|  |  | GG vs. AA | 0.001 | -0.004 | 0.007 | 0.640 |  |  |  |
|  |  | AG+GG vs. AA | 0.000 | -0.003 | 0.004 | 0.886 |  | 0.844 | -0.021 |
|  |  | GG vs. AG+AA | 0.001 | -0.003 | 0.006 | 0.598 |  | 0.782 | -0.019 |
|  | *RUNX2* rs59983488 (T/G) | GT vs. GG | 0.000 | -0.003 | 0.004 | 0.821 |  | 0.847 | -0.026 |
|  |  | TT vs. GG | 0.004 | -0.006 | 0.014 | 0.453 |  |  |  |
|  |  | GT+TT vs. GG | 0.000 | -0.003 | 0.004 | 0.705 |  | 0.814 | -0.020 |
|  |  | TT vs. GT+GG | 0.004 | -0.006 | 0.013 | 0.465 |  | 0.720 | -0.016 |
|  | *RUNX2* rs1200425 (A/G) | AG vs. GG | 0.002 | -0.002 | 0.005 | 0.374 |  | 0.713 | -0.018 |
|  |  | AA vs. GG | 0.000 | -0.005 | 0.004 | 0.811 |  |  |  |
|  |  | AG+AA vs. GG | 0.001 | -0.002 | 0.005 | 0.536 |  | 0.756 | -0.018 |
|  |  | AA vs. AG+GG | -0.002 | -0.006 | 0.003 | 0.471 |  | 0.723 | -0.016 |
|  | *SMAD6* rs2119261 (T/C) | CT vs. CC | 0.001 | -0.002 | 0.005 | 0.528 |  | 0.681 | -0.017 |
|  |  | TT vs. CC | 0.003 | -0.002 | 0.008 | 0.226 |  |  |  |
|  |  | CT+TT vs. CC | 0.002 | -0.002 | 0.005 | 0.357 |  | 0.646 | -0.013 |
|  |  | TT vs. CT+CC | 0.002 | -0.002 | 0.007 | 0.296 |  | 0.592 | -0.011 |
|  | *SMAD6* rs3934908 (T/C) | CT vs. CC | -0.003 | -0.007 | 0.000 | 0.134 |  | 0.518 | -0.007 |
|  |  | TT vs. CC | 0.000 | -0.006 | 0.004 | 0.681 |  |  |  |
|  |  | CT+TT vs. CC | -0.002 | -0.006 | 0.001 | 0.191 |  | 0.470 | -0.004 |
|  |  | TT vs. CT+CC | 0.000 | -0.003 | 0.005 | 0.681 |  | 0.808 | -0.020 |
| PC4 - Asymmetric component | *BMP2* rs1005464 (A/G) | AG vs. GG | 0.000 | -0.003 | 0.003 | 0.850 |  | 0.034* | 0.062 |
|  |  | AA vs. GG | -0.003 | -0.009 | 0.003 | 0.341 |  |  |  |
|  |  | AG+AA vs. GG | 0.000 | -0.004 | 0.002 | 0.641 |  | 0.021* | 0.065 |
|  |  | AA vs. AG+GG | -0.003 | -0.009 | 0.003 | 0.349 |  | 0.015* | 0.071 |
|  | *BMP2* rs235768 (A/T) | AT vs. TT | 0.000 | -0.003 | 0.003 | 0.983 |  | 0.015* | 0.080 |
|  |  | AA vs. TT | 0.005 | -0.001 | 0.010 | 0.109 |  |  |  |
|  |  | AT+AA vs. TT | 0.000 | -0.002 | 0.004 | 0.674 |  | 0.021* | 0.065 |
|  |  | AA vs. AT+TT | 0.005 | 0.000 | 0.010 | 0.093 |  | 0.006* | 0.089 |
|  | *BMP4* rs17563 (G/A) | AG vs. AA | -0.002 | -0.005 | 0.001 | 0.204 |  | 0.025* | 0.069 |
|  |  | GG vs. AA | -0.001 | -0.006 | 0.003 | 0.605 |  |  |  |
|  |  | AG+GG vs. AA | -0.002 | -0.005 | 0.001 | 0.226 |  | 0.012* | 0.076 |
|  |  | GG vs. AG+AA | 0.000 | -0.004 | 0.004 | 0.908 |  | 0.023* | 0.063 |
|  | *RUNX2* rs59983488 (T/G) | GT vs. GG | -0.002 | -0.005 | 0.002 | 0.319 |  | 0.029* | 0.065 |
|  |  | TT vs. GG | -0.003 | -0.012 | 0.006 | 0.533 |  |  |  |
|  |  | GT+TT vs. GG | -0.002 | -0.005 | 0.001 | 0.278 |  | 0.013* | 0.074 |
|  |  | TT vs. GT+GG | -0.002 | -0.011 | 0.007 | 0.619 |  | 0.020* | 0.065 |
|  | *RUNX2* rs1200425 (A/G) | AG vs. GG | 0.000 | -0.004 | 0.002 | 0.596 |  | 0.041* | 0.058 |
|  |  | AA vs. GG | -0.001 | -0.006 | 0.003 | 0.540 |  |  |  |
|  |  | AG+AA vs. GG | -0.001 | -0.004 | 0.002 | 0.524 |  | 0.019* | 0.067 |
|  |  | AA vs. AG+GG | 0.000 | -0.005 | 0.003 | 0.675 |  | 0.021* | 0.065 |
|  | *SMAD6* rs2119261 (T/C) | CT vs. CC | -0.002 | -0.005 | 0.001 | 0.195 |  | 0.024* | 0.070 |
|  |  | TT vs. CC | -0.002 | -0.006 | 0.003 | 0.452 |  |  |  |
|  |  | CT+TT vs. CC | -0.002 | -0.005 | 0.001 | 0.192 |  | 0.010* | 0.079 |
|  |  | TT vs. CT+CC | 0.000 | -0.004 | 0.004 | 0.832 |  | 0.022* | 0.063 |
|  | *SMAD6* rs3934908 (T/C) | CT vs. CC | 0.001 | -0.002 | 0.005 | 0.496 |  | 0.020* | 0.074 |
|  |  | TT vs. CC | -0.002 | -0.006 | 0.003 | 0.430 |  |  |  |
|  |  | CT+TT vs. CC | 0.000 | -0.003 | 0.004 | 0.802 |  | 0.022* | 0.063 |
|  |  | TT vs. CT+CC | -0.002 | -0.006 | 0.001 | 0.187 |  | 0.010* | 0.079 |
| PC5 - Asymmetric component | *BMP2* rs1005464 (A/G) | AG vs. GG | 0.000 | -0.003 | 0.003 | 0.882 |  | 0.812 | -0.024 |
|  |  | AA vs. GG | -0.003 | -0.008 | 0.003 | 0.407 |  |  |  |
|  |  | AG+AA vs. GG | 0.000 | -0.003 | 0.003 | 0.900 |  | 0.846 | -0.022 |
|  |  | AA vs. AG+GG | -0.003 | -0.008 | 0.003 | 0.383 |  | 0.667 | -0.014 |
|  | *BMP2* rs235768 (A/T) | AT vs. TT | 0.002 | 0.000 | 0.005 | 0.088 |  | 0.325 | 0.007 |
|  |  | AA vs. TT | 0.004 | -0.002 | 0.009 | 0.166 |  |  |  |
|  |  | AT+AA vs. TT | 0.003 | 0.000 | 0.005 | 0.057 |  | 0.216 | 0.015 |
|  |  | AA vs. AT+TT | 0.002 | -0.003 | 0.008 | 0.344 |  | 0.637 | -0.013 |
|  | *BMP4* rs17563 (G/A) | AG vs. AA | 0.001 | -0.002 | 0.004 | 0.488 |  | 0.863 | -0.027 |
|  |  | GG vs. AA | 0.000 | -0.004 | 0.005 | 0.804 |  |  |  |
|  |  | AG+GG vs. AA | 0.001 | -0.002 | 0.004 | 0.514 |  | 0.747 | -0.017 |
|  |  | GG vs. AG+AA | 0.000 | -0.004 | 0.004 | 0.919 |  | 0.848 | -0.022 |
|  | *RUNX2* rs59983488 (T/G) | GT vs. GG | 0.000 | -0.004 | 0.002 | 0.577 |  | 0.859 | -0.027 |
|  |  | TT vs. GG | -0.002 | -0.010 | 0.006 | 0.604 |  |  |  |
|  |  | GT+TT vs. GG | 0.000 | -0.004 | 0.002 | 0.519 |  | 0.749 | -0.017 |
|  |  | TT vs. GT+GG | -0.002 | -0.010 | 0.006 | 0.652 |  | 0.801 | -0.020 |
|  | *RUNX2* rs1200425 (A/G) | AG vs. GG | -0.002 | -0.005 | 0.001 | 0.299 |  | 0.690 | -0.017 |
|  |  | AA vs. GG | -0.002 | -0.006 | 0.002 | 0.315 |  |  |  |
|  |  | AG+AA vs. GG | -0.002 | -0.005 | 0.001 | 0.239 |  | 0.532 | -0.008 |
|  |  | AA vs. AG+GG | -0.001 | -0.005 | 0.003 | 0.548 |  | 0.762 | -0.018 |
|  | *SMAD6* rs2119261 (T/C) | CT vs. CC | 0.000 | -0.003 | 0.003 | 0.964 |  | 0.938 | -0.032 |
|  |  | TT vs. CC | 0.000 | -0.004 | 0.004 | 0.909 |  |  |  |
|  |  | CT+TT vs. CC | 0.000 | -0.003 | 0.003 | 0.940 |  | 0.849 | -0.022 |
|  |  | TT vs. CT+CC | 0.000 | -0.004 | 0.004 | 0.917 |  | 0.847 | -0.022 |
|  | *SMAD6* rs3934908 (T/C) | CT vs. CC | -0.004 | -0.007 | 0.000 | 0.016* |  | 0.154 | 0.026 |
|  |  | TT vs. CC | -0.003 | -0.007 | 0.001 | 0.177 |  |  |  |
|  |  | CT+TT vs. CC | -0.004 | -0.006 | 0.000 | 0.019* |  | 0.097 | 0.033 |
|  |  | TT vs. CT+CC | 0.000 | -0.004 | 0.003 | 0.896 |  | 0.846 | -0.022 |
| PC6 - Asymmetric component | *BMP2* rs1005464 (A/G) | AG vs. GG | 0.000 | -0.003 | 0.003 | 0.975 |  | 0.447 | -0.003 |
|  |  | AA vs. GG | 0.003 | -0.002 | 0.009 | 0.252 |  |  |  |
|  |  | AG+AA vs. GG | 0.000 | -0.002 | 0.003 | 0.700 |  | 0.474 | -0.005 |
|  |  | AA vs. AG+GG | 0.003 | -0.002 | 0.008 | 0.244 |  | 0.293 | 0.007 |
|  | *BMP2* rs235768 (A/T) | AT vs. TT | 0.000 | -0.002 | 0.003 | 0.592 |  | 0.520 | -0.007 |
|  |  | AA vs. TT | 0.002 | -0.003 | 0.007 | 0.377 |  |  |  |
|  |  | AT+AA vs. TT | 0.000 | -0.002 | 0.003 | 0.471 |  | 0.410 | 0.000 |
|  |  | AA vs. AT+TT | 0.002 | -0.003 | 0.006 | 0.441 |  | 0.398 | 0.000 |
|  | *BMP4* rs17563 (G/A) | AG vs. AA | 0.000 | -0.003 | 0.002 | 0.743 |  | 0.487 | -0.005 |
|  |  | GG vs. AA | -0.002 | -0.006 | 0.002 | 0.308 |  |  |  |
|  |  | AG+GG vs. AA | 0.000 | -0.004 | 0.002 | 0.579 |  | 0.445 | -0.003 |
|  |  | GG vs. AG+AA | -0.002 | -0.005 | 0.002 | 0.322 |  | 0.341 | 0.004 |
|  | *RUNX2* rs59983488 (T/G) | GT vs. GG | 0.001 | -0.001 | 0.004 | 0.336 |  | 0.395 | 0.001 |
|  |  | TT vs. GG | -0.003 | -0.010 | 0.005 | 0.450 |  |  |  |
|  |  | GT+TT vs. GG | 0.000 | -0.002 | 0.004 | 0.454 |  | 0.403 | 0.000 |
|  |  | TT vs. GT+GG | -0.003 | -0.011 | 0.004 | 0.373 |  | 0.368 | 0.002 |
|  | *RUNX2* rs1200425 (A/G) | AG vs. GG | 0.000 | -0.003 | 0.003 | 0.969 |  | 0.551 | -0.009 |
|  |  | AA vs. GG | -0.001 | -0.005 | 0.002 | 0.472 |  |  |  |
|  |  | AG+AA vs. GG | 0.000 | -0.003 | 0.002 | 0.824 |  | 0.492 | -0.006 |
|  |  | AA vs. AG+GG | -0.001 | -0.005 | 0.002 | 0.406 |  | 0.383 | 0.000 |
|  | *SMAD6* rs2119261 (T/C) | CT vs. CC | 0.001 | -0.001 | 0.004 | 0.290 |  | 0.462 | -0.004 |
|  |  | TT vs. CC | 0.002 | -0.002 | 0.005 | 0.437 |  |  |  |
|  |  | CT+TT vs. CC | 0.001 | -0.001 | 0.004 | 0.263 |  | 0.305 | 0.006 |
|  |  | TT vs. CT+CC | 0.000 | -0.003 | 0.004 | 0.723 |  | 0.478 | -0.005 |
|  | *SMAD6* rs3934908 (T/C) | CT vs. CC | 0.000 | -0.002 | 0.004 | 0.675 |  | 0.426 | -0.001 |
|  |  | TT vs. CC | 0.002 | -0.001 | 0.006 | 0.231 |  |  |  |
|  |  | CT+TT vs. CC | 0.001 | -0.002 | 0.004 | 0.456 |  | 0.404 | 0.000 |
|  |  | TT vs. CT+CC | 0.002 | -0.001 | 0.005 | 0.251 |  | 0.297 | 0.007 |
| PC7 - Asymmetric component | *BMP2* rs1005464 (A/G) | AG vs. GG | 0.000 | -0.003 | 0.002 | 0.572 |  | 0.752 | -0.021 |
|  |  | AA vs. GG | 0.000 | -0.005 | 0.005 | 0.907 |  |  |  |
|  |  | AG+AA vs. GG | 0.000 | -0.003 | 0.002 | 0.637 |  | 0.621 | -0.012 |
|  |  | AA vs. AG+GG | 0.000 | -0.005 | 0.006 | 0.825 |  | 0.661 | -0.014 |
|  | *BMP2* rs235768 (A/T) | AT vs. TT | 0.000 | -0.002 | 0.003 | 0.921 |  | 0.757 | -0.021 |
|  |  | AA vs. TT | 0.001 | -0.003 | 0.006 | 0.561 |  |  |  |
|  |  | AT+AA vs. TT | 0.000 | -0.002 | 0.003 | 0.803 |  | 0.658 | -0.014 |
|  |  | AA vs. AT+TT | 0.001 | -0.003 | 0.006 | 0.562 |  | 0.597 | -0.011 |
|  | *BMP4* rs17563 (G/A) | AG vs. AA | 0.001 | 0.000 | 0.004 | 0.213 |  | 0.368 | 0.003 |
|  |  | GG vs. AA | 0.003 | 0.000 | 0.007 | 0.121 |  |  |  |
|  |  | AG+GG vs. AA | 0.002 | 0.000 | 0.005 | 0.142 |  | 0.293 | 0.007 |
|  |  | GG vs. AG+AA | 0.002 | -0.002 | 0.005 | 0.278 |  | 0.435 | -0.002 |
|  | *RUNX2* rs59983488 (T/G) | GT vs. GG | 0.001 | -0.001 | 0.004 | 0.404 |  | 0.690 | -0.017 |
|  |  | TT vs. GG | 0.000 | -0.007 | 0.007 | 0.962 |  |  |  |
|  |  | GT+TT vs. GG | 0.000 | -0.001 | 0.003 | 0.420 |  | 0.532 | -0.008 |
|  |  | TT vs. GT+GG | 0.000 | -0.007 | 0.007 | 0.949 |  | 0.671 | -0.014 |
|  | *RUNX2* rs1200425 (A/G) | AG vs. GG | 0.000 | -0.003 | 0.002 | 0.865 |  | 0.778 | -0.022 |
|  |  | AA vs. GG | 0.000 | -0.003 | 0.004 | 0.747 |  |  |  |
|  |  | AG+AA vs. GG | 0.000 | -0.003 | 0.003 | 0.984 |  | 0.672 | -0.014 |
|  |  | AA vs. AG+GG | 0.000 | -0.003 | 0.004 | 0.653 |  | 0.626 | -0.012 |
|  | *SMAD6* rs2119261 (T/C) | CT vs. CC | 0.000 | -0.003 | 0.002 | 0.691 |  | 0.731 | -0.019 |
|  |  | TT vs. CC | 0.000 | -0.003 | 0.004 | 0.732 |  |  |  |
|  |  | CT+TT vs. CC | 0.000 | -0.003 | 0.002 | 0.838 |  | 0.662 | -0.014 |
|  |  | TT vs. CT+CC | 0.000 | -0.002 | 0.004 | 0.567 |  | 0.599 | -0.011 |
|  | *SMAD6* rs3934908 (T/C) | CT vs. CC | 0.000 | -0.004 | 0.002 | 0.548 |  | 0.136 | 0.030 |
|  |  | TT vs. CC | -0.004 | -0.007 | 0.000 | 0.025* |  |  |  |
|  |  | CT+TT vs. CC | -0.002 | -0.004 | 0.000 | 0.216 |  | 0.378 | 0.001 |
|  |  | TT vs. CT+CC | -0.003 | -0.006 | 0.000 | 0.024* |  | 0.084 | 0.036 |
| Log centroid size | *BMP2* rs1005464 (A/G) | AG vs. GG | 0.014 | 0.000 | 0.026 | 0.036* |  | <0.001* | 0.498 |
|  |  | AA vs. GG | 0.022 | -0.004 | 0.047 | 0.090 |  |  |  |
|  |  | AG+AA vs. GG | 0.015 | 0.003 | 0.027 | 0.016* |  | <0.001* | 0.501 |
|  |  | AA vs. AG+GG | 0.017 | -0.008 | 0.042 | 0.186 |  | <0.001* | 0.481 |
|  | *BMP2* rs235768 (A/T) | AT vs. TT | -0.010 | -0.022 | 0.003 | 0.123 |  | <0.001* | 0.479 |
|  |  | AA vs. TT | -0.003 | -0.026 | 0.020 | 0.815 |  |  |  |
|  |  | AT+AA vs. TT | -0.009 | -0.021 | 0.003 | 0.150 |  | <0.001* | 0.482 |
|  |  | AA vs. AT+TT | 0.002 | -0.020 | 0.025 | 0.845 |  | <0.001* | 0.472 |
|  | *BMP4* rs17563 (G/A) | AG vs. AA | -0.001 | -0.015 | 0.013 | 0.871 |  | <0.001* | 0.467 |
|  |  | GG vs. AA | 0.002 | -0.018 | 0.021 | 0.862 |  |  |  |
|  |  | AG+GG vs. AA | 0.000 | -0.014 | 0.013 | 0.929 |  | <0.001* | 0.472 |
|  |  | GG vs. AG+AA | 0.003 | -0.015 | 0.020 | 0.772 |  | <0.001* | 0.472 |
|  | *RUNX2* rs59983488 (T/G) | GT vs. GG | -0.004 | -0.017 | 0.009 | 0.520 |  | <0.001* | 0.470 |
|  |  | TT vs. GG | -0.012 | -0.049 | 0.024 | 0.498 |  |  |  |
|  |  | GT+TT vs. GG | -0.005 | -0.017 | 0.008 | 0.447 |  | <0.001* | 0.475 |
|  |  | TT vs. GT+GG | -0.011 | -0.047 | 0.025 | 0.548 |  | <0.001* | 0.473 |
|  | *RUNX2* rs1200425 (A/G) | AG vs. GG | -0.002 | -0.015 | 0.012 | 0.813 |  | <0.001* | 0.468 |
|  |  | AA vs. GG | -0.005 | -0.023 | 0.013 | 0.587 |  |  |  |
|  |  | AG+AA vs. GG | -0.002 | -0.015 | 0.010 | 0.704 |  | <0.001* | 0.472 |
|  |  | AA vs. AG+GG | -0.004 | -0.020 | 0.012 | 0.624 |  | <0.001* | 0.473 |
|  | *SMAD6* rs2119261 (T/C) | CT vs. CC | 0.001 | -0.012 | 0.015 | 0.846 |  | <0.001* | 0.467 |
|  |  | TT vs. CC | -0.002 | -0.020 | 0.017 | 0.840 |  |  |  |
|  |  | CT+TT vs. CC | 0.000 | -0.012 | 0.013 | 0.930 |  | <0.001* | 0.472 |
|  |  | TT vs. CT+CC | -0.003 | -0.019 | 0.014 | 0.750 |  | <0.001* | 0.472 |
|  | *SMAD6* rs3934908 (T/C) | CT vs. CC | 0.005 | -0.009 | 0.019 | 0.497 |  | <0.001* | 0.474 |
|  |  | TT vs. CC | 0.011 | -0.007 | 0.028 | 0.243 |  |  |  |
|  |  | CT+TT vs. CC | 0.006 | -0.007 | 0.020 | 0.348 |  | <0.001* | 0.476 |
|  |  | TT vs. CT+CC | 0.007 | -0.008 | 0.023 | 0.337 |  | <0.001* | 0.476 |
| Mahalanobis shape FA scores | *BMP2* rs1005464 (A/G) | AG vs. GG | 0.049 | -0.265 | 0.363 | 0.756 |  | 0.412 | 0.000 |
|  |  | AA vs. GG | -0.520 | -1.155 | 0.116 | 0.108 |  |  |  |
|  |  | AG+AA vs. GG | -0.034 | -0.336 | 0.268 | 0.823 |  | 0.796 | -0.019 |
|  |  | AA vs. AG+GG | -0.537 | -1.159 | 0.085 | 0.090 |  | 0.275 | 0.009 |
|  | *BMP2* rs235768 (A/T) | AT vs. TT | 0.320 | 0.017 | 0.623 | 0.039* |  | 0.257 | 0.013 |
|  |  | AA vs. TT | 0.169 | -0.394 | 0.732 | 0.553 |  |  |  |
|  |  | AT+AA vs. TT | 0.298 | 0.007 | 0.589 | 0.044* |  | 0.168 | 0.020 |
|  |  | AA vs. AT+TT | 0.006 | -0.545 | 0.557 | 0.982 |  | 0.808 | -0.020 |
|  | *BMP4* rs17563 (G/A) | AG vs. AA | -0.113 | -0.449 | 0.223 | 0.507 |  | 0.473 | -0.004 |
|  |  | GG vs. AA | 0.230 | -0.250 | 0.709 | 0.344 |  |  |  |
|  |  | AG+GG vs. AA | -0.049 | -0.378 | 0.279 | 0.767 |  | 0.786 | -0.019 |
|  |  | GG vs. AG+AA | 0.308 | -0.108 | 0.726 | 0.147 |  | 0.376 | 0.001 |
|  | *RUNX2* rs59983488 (T/G) | GT vs. GG | -0.244 | -0.552 | 0.065 | 0.121 |  | 0.324 | 0.007 |
|  |  | TT vs. GG | -0.590 | -1.473 | 0.292 | 0.188 |  |  |  |
|  |  | GT+TT vs. GG | -0.269 | -0.570 | 0.032 | 0.079 |  | 0.252 | 0.011 |
|  |  | TT vs. GT+GG | -0.499 | -1.380 | 0.383 | 0.264 |  | 0.526 | -0.007 |
|  | *RUNX2* rs1200425 (A/G) | AG vs. GG | -0.055 | -0.388 | 0.278 | 0.744 |  | 0.768 | -0.021 |
|  |  | AA vs. GG | -0.206 | -0.652 | 0.240 | 0.361 |  |  |  |
|  |  | AG+AA vs. GG | -0.093 | -0.409 | 0.223 | 0.561 |  | 0.726 | -0.017 |
|  |  | AA vs. AG+GG | -0.173 | -0.568 | 0.223 | 0.388 |  | 0.631 | -0.012 |
|  | *SMAD6* rs2119261 (T/C) | CT vs. CC | 0.129 | -0.198 | 0.455 | 0.436 |  | 0.792 | -0.023 |
|  |  | TT vs. CC | 0.145 | -0.308 | 0.597 | 0.528 |  |  |  |
|  |  | CT+TT vs. CC | 0.132 | -0.177 | 0.442 | 0.398 |  | 0.638 | -0.013 |
|  |  | TT vs. CT+CC | 0.068 | -0.340 | 0.475 | 0.742 |  | 0.781 | -0.019 |
|  | *SMAD6* rs3934908 (T/C) | CT vs. CC | -0.144 | -0.486 | 0.198 | 0.407 |  | 0.581 | -0.011 |
|  |  | TT vs. CC | 0.115 | -0.323 | 0.552 | 0.605 |  |  |  |
|  |  | CT+TT vs. CC | -0.076 | -0.403 | 0.251 | 0.645 |  | 0.756 | -0.018 |
|  |  | TT vs. CT+CC | 0.208 | -0.168 | 0.584 | 0.275 |  | 0.536 | -0.008 |

SNP – single nucleotide polymorphism, CI – confidence interval, PC – principal component, Log – natural logarithm, FA – fluctuating asymmetry.

† (1 = minor allele / 2 = major allele).

* Indicates statistical significance.
